# Dual deep modeling: multi-level modeling with dual potencies and its formalization in F-Logic

**DOI:** 10.1007/s10270-016-0519-z

**Published:** 2016-04-07

**Authors:** Bernd Neumayr, Christoph G. Schuetz, Manfred A. Jeusfeld, Michael Schrefl

**Affiliations:** 1 Johannes Kepler University Linz, Linz, Austria; 2 University of Skövde, Skövde, Sweden

**Keywords:** Database modeling, Deep instantiation, Clabject, UML, Deep modeling notation

## Abstract

An enterprise database contains a global, integrated, and consistent representation of a company’s data. Multi-level modeling facilitates the definition and maintenance of such an integrated conceptual data model in a dynamic environment of changing data requirements of diverse applications. Multi-level models transcend the traditional separation of class and object with clabjects as the central modeling primitive, which allows for a more flexible and natural representation of many real-world use cases. In deep instantiation, the number of instantiation levels of a clabject or property is indicated by a single potency. Dual deep modeling (DDM) differentiates between source potency and target potency of a property or association and supports the flexible instantiation and refinement of the property by statements connecting clabjects at different modeling levels. DDM comes with multiple generalization of clabjects, subsetting/specialization of properties, and multi-level cardinality constraints. Examples are presented using a UML-style notation for DDM together with UML class and object diagrams for the representation of two-level user views derived from the multi-level model. Syntax and semantics of DDM are formalized and implemented in F-Logic, supporting the modeler with integrity checks and rich query facilities.

## Introduction

According to the widely accepted ANSI/SPARC architecture [21], an enterprise database serves data to different applications through different *user views* defined on top of the *conceptual data model* which abstracts from the internal representation of data. In this setting, the conceptual data model is a global, integrated, redundancy-free, and consistent representation of a company’s data. The user views are application-specific extracts from the conceptual data model.

Object orientation is arguably the most popular paradigm for conceptual data modeling. In this paradigm, class and object are the two central model elements. In real-world settings, however, the boundaries between what is represented as class and what is represented as object are rather fluid. What acts as object in one application may act as class in another application. For example, in an application for managing a catalog of cars, the Car class is instantiated by objects representing particular car models, such as Porsche911. In an application for managing an inventory of serviced cars, the same car model may be represented by the Porsche911 class, which is instantiated by objects representing individual cars, such as MarysCar.

The multi-level approach to conceptual data modeling transcends the separation of class and object from traditional two-level object-oriented modeling. A *multi-level conceptual model* employs the notion of *clabject*, which may act as class and object alike. In a multi-level conceptual model, a single model element, e.g., the Porsche911 clabject, may represent both an individual object, e.g., the Porsche911 car model, and a class of objects, e.g., the ensemble of individual Porsche911 cars.

Multi-level modeling facilitates the definition and maintenance of a conceptual data model. First, clabjects accurately reflect the dual nature of many real-world entities represented as class in one application and as object in another application, which facilitates consideration of different data demands from various applications in a single conceptual data model. Furthermore, in a dynamic business environment, companies are confronted with constantly changing market demands, which must be reflected accordingly in the applications and the data used by these applications. Related work [33] demonstrates that the dynamic introduction and manipulation of types are more easily accomplished in multi-level models.

**Fig. 1:**
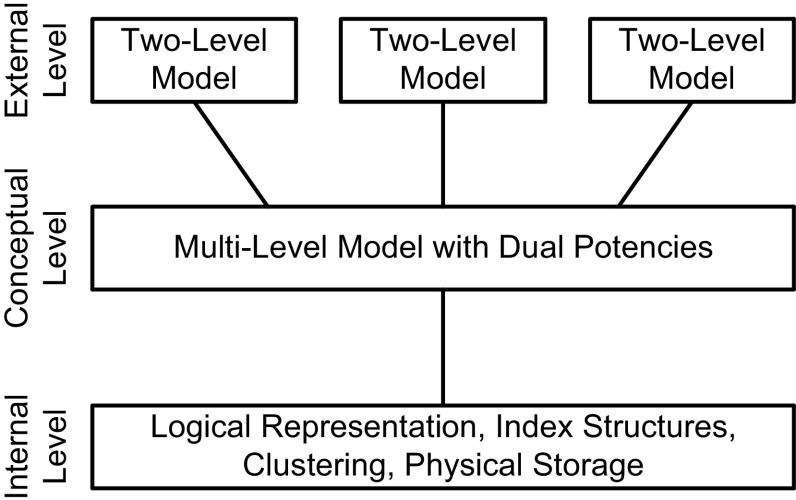
Multi-level modeling with dual potencies in the ANSI/SPARC architecture

In accordance with the ANSI/SPARC architecture, applications may use traditional two-level views for accessing the data represented by the multi-level conceptual model (Fig. 1). *Dual Deep Models*, i.e., multi-level models with dual potencies, may serve as integrated conceptual data models at the conceptual level of the ANSI/SPARC architecture, whereas traditional UML class and object diagrams, i.e., two-level models, may serve as the representation of user views at the external level.

In this paper, we introduce dual deep modeling (DDM) as a general purpose approach to multi-level modeling geared especially toward conceptual data modeling. We concentrate on the conceptual level of the ANSI/SPARC architecture (Fig. 1). We abstract from the internal database level and only sketch the definition of two-level views on top of a DDM-based conceptual model.

Conceptual data models present certain particularities when compared to models in software engineering. First, a database may contain incomplete data, e.g., NULL values in relational databases. Second, a conceptual data model typically does not prescribe the navigability of a relationship. Rather, relationships can be queried and constrained in both directions. Third, a conceptual database model requires rich constructs for cardinality constraints, which in turn require clear principles of how to count clabjects. Finally, a database always comes with rich query facilities. The definition of DDM takes into account these particularities.

The structure and semantics of DDM constructs are formalized in the Flora-2 [46] variant of F-Logic [25], a mature modeling language with concise syntax as well as metamodeling capabilities. The F-Logic formalization of the DDM approach, referred to as DDM-FL, together with the Flora-2 system, provides a proof-of-concept implementation which automates (i) structural integrity checks, (ii) derivation of range and values of a property which takes into account higher-level statements, multiple clabject generalization, property specialization, and inverse properties. The proof-of-concept implementation supports the modeling process and provides the query facilities required in a database setting.

DDM as presented in this paper considerably extends our previous work on dual deep instantiation (DDI [36]). The underlying axioms allow for more expressive models featuring multiple inheritance, the possibility of concurrent use of instantiation and specialization, deep cardinality constraints, and property specialization. DDM incorporates ideas, namely the abstract super-clabject rule and deep mandatory constraints, which were first presented and formalized in the setting of a simplified variant [37] of DDI only supporting unidirectional and single-valued properties. Furthermore, DDM introduces query facilities for range, value, and active range. The formalization in F-Logic provides a more elegant means for working with DDM models. The embedding of DDM in an ANSI/SPARC setting, with the possibility of traditional two-level user views from a multi-level integrated conceptual data model, ensures interoperability with legacy applications.

The remainder of this paper is organized as follows: In Sect. 2, we present DDM in a nutshell. In Sect. 3, we introduce clabject hierarchies and properties with dual potencies. In Sect. 4, we discuss the instance-level and schema-level facet of statements, i.e., *value* and *range*, respectively. In Sect. 5, we discuss the generalization of clabjects. In Sect. 6, we discuss the specialization of properties. In Sect. 7, we discuss cardinality constraints. In Sect. 8, we review related work. Section 9 concludes the paper with an outlook on future work.

## Overview

**Fig. 2:**
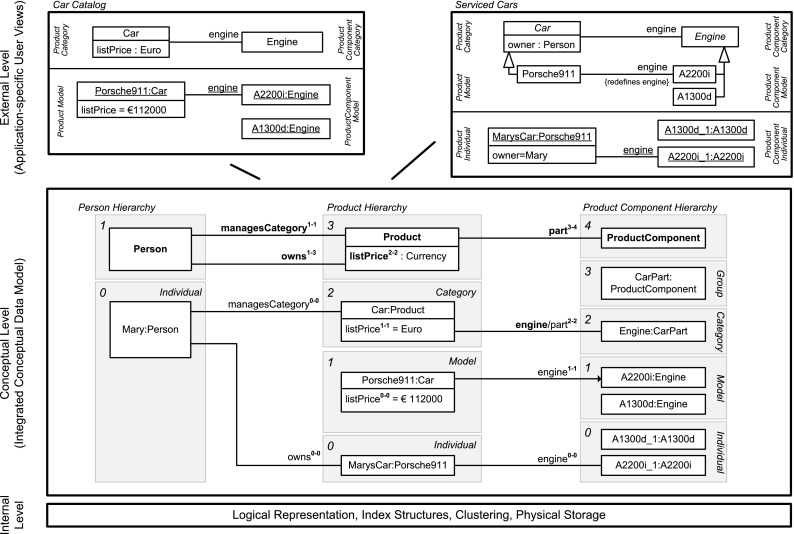
A fragment of the enterprise database of the fictional ACME corporation. Dual deep modeling is used at the conceptual level. Users and applications interact with the database through two-level user views

In this section, we sketch the essence of DDM, using the DDM model in Fig. 2 for illustration. In the remainder of this paper, we then provide a detailed presentation of the DDM modeling constructs in a stepwise manner accompanied by a discussion of the background from two-level modeling, examples with a graphical notation as well as a formalization and implementation in F-Logic. Table 1 gives an overview of the main elements of the graphical notation and contains references to positions in the remainder of this paper with a more detailed presentation of the corresponding model elements.

The conceptual layer of the ANSI/SPARC architecture may contain a DDM model as integrated conceptual data model, whereas the external level may contain two-level representations derived from the DDM model as user views. The integrated conceptual data model in Fig. 2 consists of hierarchical representations of persons, products, and product components. Each white box represents a clabject. The clabjects of the different hierarchies are related by deep properties. The different user views then emphasize different levels of the clabject hierarchies from the integrated conceptual model, limiting the multi-level model to a traditional two-level model with class and objects. For example, the conceptual model contains information about product categories, models in these categories, and individual products, i.e., physical entities. The car catalog view then focuses on the *Model* level of the product hierarchy. The serviced cars view, on the other hand, focuses on the *Individual* level of the product hierarchy.

In contrast to strict metamodeling [7], DDM allows to specify different clabject hierarchies with a different number of instantiation levels. For example, Product is specified with three instantiation levels, namely product categories, e.g., Car, which are instantiated by product models, e.g., Porsche911, which are in turn instantiated by product individuals, e.g., MarysCar. Person only has a single instantiation level, with individual person Mary as an instance.

Dual potencies extend deep instantiation with the possibility to indicate the depth of characterization separately for the source and the target of a property. For example, property owns with source clabject Person and target clabject Product has source potency 1 and target potency 3, which means that this property is instantiated by statements relating individual persons and individual products, e.g., ‘Mary owns MarysCar’. On the other hand, property managesCategory has target potency 1, which means that this property is instantiated by statements relating individual persons and product categories, e.g., ‘Mary managesCategory Car’. We further discuss how dual potencies are used to model self-describing objects, thereby generalizing the notion of *singleton class* to multi-level modeling.

**Table 1:**
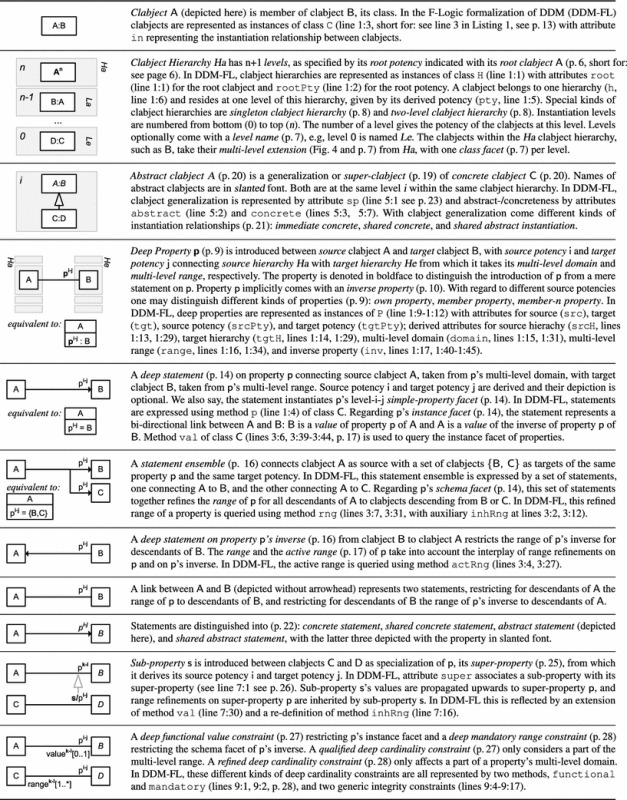
The DDM notation—with references to the main concepts and their formalization in F-Logic

A statement at a higher level acts as property value at that level and as property range at lower levels. For example, the higher-level statement ‘Porsche911 engine A2200i’ is first regarded as property value (represented in user view *Car Catalog* as link between objects Porsche911 and A2200i) and second as range refinement at lower modeling levels (represented in user view *Serviced Cars* as an association between classes Porsche911 and A2200i that redefines association end engine). We further discuss the handling of incomplete data (missing statements, NULL values) and multi-valued properties (M:N relationships) and their interpretation regarding range refinement at lower levels.

We introduce inverse properties as a means to extend properties to bidirectional relationships as required for conceptual data modeling. Every property implicitly has an inverse property that can be addressed in queries and in statements. For example, the inverse of property owns is represented in user view *Serviced Cars* by attribute owner. In the paper, we discuss the interplay between statements in different directions (i.e., on a property and on its inverse property). We will show how to derive bidirectional links (values of property and of its inverse property) and associations (range of a property and of its inverse property) from unidirectional statements.

Clabjects may generalize a set of other clabjects. The strict separation between concrete clabjects and abstract super-clabjects [37] provides a clear principle for counting clabjects. Multiple generalization as well as the interplay between instantiation and generalization are other aspects addressed in this paper.

The specialization of properties—also referred to as subsetting of properties—allows for the definition of hierarchies of properties as well as the separate instantiation and refinement of different sub-properties. For example, property part between clabjects Product and ProductComponent is specialized by property engine. The interplay between sub-properties and super-properties regarding range refinement and querying is also addressed in this paper.

Modelers may specify cardinality constraints at different modeling levels. More specifically, properties may be marked functional and/or mandatory. Dual deep cardinality constraints allow modelers to constrain the number of property targets, either regarding property range or property values, between instances of a given source clabject and instances of a given target clabject for a given target potency and source potency. In the paper, we also discuss the different kinds of cardinality constraints made possible by different combinations of these parameters.

**Fig. 3:**
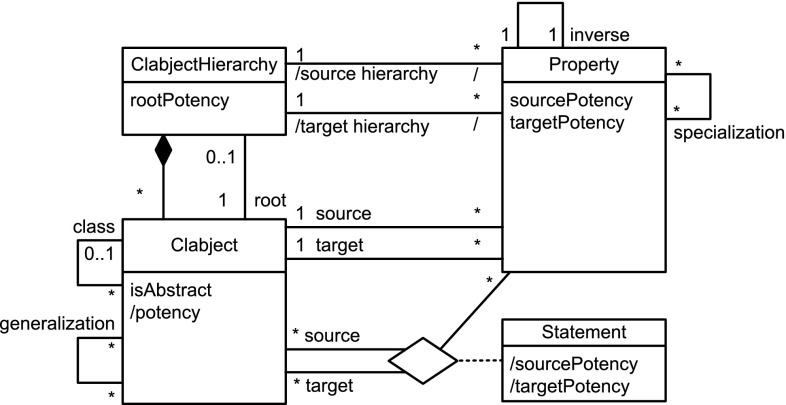
Linguistic metamodel of the DDM approach

The linguistic metamodel of DDM (Fig. 3) gives a concise overview of the structure of dual deep models. Clabjects are organized in clabject hierarchies with a single root clabject and a root potency. The instantiation relationship between clabjects determines the hierarchical order of the clabjects. A clabject may also specialize several other clabjects, which are referred to as the generalizations of the specializing clabject. A property links a source clabject with a target clabject, and therefore, it also links a source hierarchy with a target hierarchy and has a specific source and target potency. A property may specialize several other properties. Each property has an inverse property. A triple of source clabject, target clabject, and property constitutes a statement with a derived source potency and a derived target potency.

## Clabjects and dual deep properties

Dual deep modeling (DDM) is an object-oriented multi-level modeling approach. As a background, we first clarify our understanding of some basic notions of two-level object-oriented modeling, where classes are instantiated by objects, and properties are instantiated by statements. We then extend these basic notions to DDM, where clabjects are organized in multi-level instantiation hierarchies and characterized and connected by deep properties. All DDM constructs are formalized in F-Logic with example dual deep models depicted in an UML-like graphical notation and in F-Logic.

### Background: classes, objects, properties, and statements

We first explain the two-level modeling constructs that we extend in the following to multi-level modeling constructs.

A *class* has a dual role, and it defines, first, the common structure or schema of its instances (structural facet), and, second, it is a container for the set of its instances (extensional facet). The set of instances of a class is referred to as the class’ extension. An *object* is instance of exactly one *class*, i.e., it instantiates the structure introduced with the class, and it is member of the class (more exactly, it is a member of the class’ extension).

Classes are characterized by their properties (attributes and association ends). The structure of objects in terms of properties is introduced with the classes. A *property* associates two classes, one acting as *source* (or domain) and the other as *target* (or range) of the property. The schema of a class is given by the set of properties that have the class as domain. A property is instantiated by statements. A statement links two objects, and we refer to them as the *owner* (or *source*) and the *value* (or *target*) of the statement. The source of a statement must be taken from the domain of the property, given by the extension of the source class. The target of a statement must be taken from the range of the property, given by the extension of the target class. An object is characterized by the set of statements it owns.

A property may have an *inverse property*, also referred to as *opposite*, which is used to navigate statements from target to source. A property together with its inverse property makes up a binary, bidirectional association, or relationship type. The inverse property may be explicitly specified in the model, e.g., by specifying role names for both ends of an association, or be given implicitly. For example, an association ownership has two association ends represented by properties owner and owns.

For simplicity and conciseness, we represent a binary association by one named property, e.g., owner, and do not explicitly represent association names or names of inverse properties (they instead get a derived name, e.g., inv(owner) instead of owns).

**Fig. 4:**
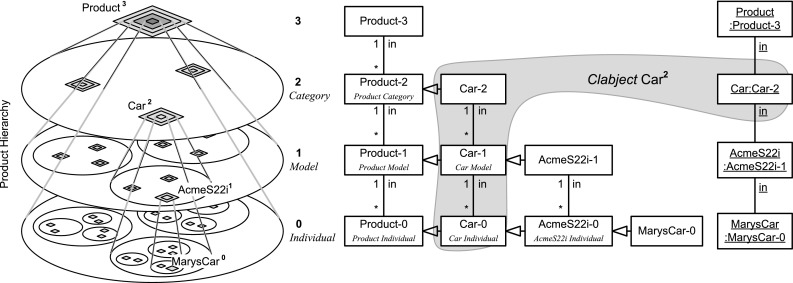
*Left* Visualization of a product hierarchy with four instantiation levels. Each level can be addressed by a number indicating the potency of the clabjects that reside at that level, or alternatively by an intuitive level name (shown in *italics*). *Right* UML representation of the object facet and the different class facets of clabject Car with potency 2 and its class Product at level 3 and its members AcmeS22i at level 1 and MarysCar at level 0. Class Car-2 is the singleton class of object Car. Alternative class names such as *CarModel* and *CarIndividual* are shown in *italics*

In his seminal work on the entity-relationship model, Chen [10] avoids modeling with primitive data types, such as Integer, but instead promotes *value sets*, e.g., NrOfYears, as a semantically richer alternative. We follow this approach and use the notion of *semantic data type* synonymously with *value set*. Semantic data types, e.g., NrOfYears and LengthInKm, are instantiated by *semantic data values*, e.g., 17 years and 23 km. For simplicity and conciseness, we do not distinguish between semantic data values/types and proper objects/classes but uniformly treat them as objects and classes. We further do not distinguish between attributes and associations but uniformly represent them as a property with an inverse property.

A *self-describing object* is an object that defines its own schema by introducing its own properties. For example, in the programming language Ruby, one may introduce properties not only with a class but also with an object, thereby defining or extending the schema of the object. In Ruby, this is accomplished by implicitly introducing with an object a singleton class, a so-called eigenclass.

A *member property of a class* is propagated to its members of which it is an own property. An *own property of a class* is a property that is not propagated to its instances but instantiated by the class itself. An own property of a class, unlike a member property, describes the class and not its instances. A class that has own properties can be considered as an object.

### Modeling clabject hierarchies

In this subsection, we discuss the basic intuition underlying clabject hierarchies, the structure of clabject hierarchies, and discuss some special kinds of clabject hierarchies.

#### Identifying kinds of objects and their instantiation levels

The first step in creating a multi-level model in DDM is to identify the kinds of objects, such as *persons*, *products*, and *product components*, to be represented and to identify the instantiation levels separately for each kind of objects (such as instantiation levels *product category*, *product model*, and *product individual* for products).

Every instantiation level comes with its principle of identity, i.e., a principle that allows to decide whether two objects at this level are different or the same, and thus facilitates the counting of objects at this level, which is a prerequisite for cardinality constraints. Every combination of clabject at some level and instantiation level below can be regarded as a class. Instantiation levels are also referred to in this paper as modeling levels, levels of identity, or classification levels. Instantiation levels are by design (up to the modeler) or represent a (possibly already long lasting and organizationally implemented) shared conceptualization of the domain of interest.

##### *Example 1*

(Different levels for different kinds of objects—Fig. 4) Objects in the domain of interest of the information system of the fictional ACME corporation include *persons*, *products*, and *product components*. Persons are modeled at a single level of identity containing individual persons like *Mary* and *MsBlack*. As typical for artificial and designed objects, products and product components have multiple levels of identity. The instantiation levels of products are *individual*, *model*, and *category*. Individual products (physical entities such as *Mary’s Car*) are instances of product models (such as car model *AcmeS22i*) which are in turn members of product categories (such as *Car*). This multi-level product hierarchy is visualized at the left of Fig. 4. The objects at higher levels (such as car model *AcmeS22i* or product category *Car*) are not mere collections of objects at lower levels but have their own properties and can be counted. The instantiation levels of *product components* are similar, with an additional level *product component group* above category, reflecting the organizational structure of the ACME corporation which is organized along these product component groups with one development group and one head of development for each group of product components. A product component group like *special equipment* contains product component categories such as *alarm* and *cruise control*.

#### Structure of clabject hierarchies

In DDM, a kind of objects with all its abstraction levels is represented by a *clabject hierarchy*. The number of instantiation levels is represented by the hierarchy’s *root potency* which is also the potency of its single *root clabject*. A clabject hierarchy with potency *n* has $n+1$ levels (including its top level). The instantiation levels of a hierarchy are numbered from top (starting with the root potency) to bottom (with potency 0). These level numbers indicate the potency of the clabjects at these levels.

##### *Example 2*

**Fig. 5:**
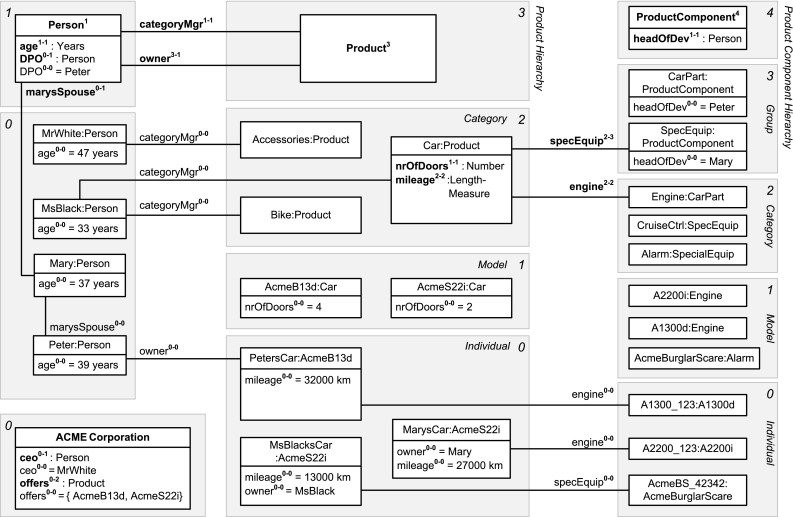
A fragment of the conceptual model of the ACME enterprise database. Clabject hierarchies and deep properties are shown in an UML-style notation. For presentation purposes (without semantic differences), some of the property declarations and statements are depicted as attributes and some as associations/links

(Multi-level clabject hierarchies—Fig. 5) *Products* and *product components* are represented by clabject hierarchies with root clabject Product with potency 3, and ProductComponent with potency 4, respectively.

A clabject in a multi-level clabject hierarchy may have members at multiple levels and be member of multiple classes, one for each higher level. A clabject *c* with potency *n* in (i.e., at level *n* of) a clabject hierarchy with root potency *m* (i.e., a clabject hierarchy with $m+1$ levels), with $n \le m$, has members at the levels beneath itself, from level 0 to level *n* (regarding the clabject itself as a member of its singleton class facet), and we refer to these different sets of clabjects as the *multi-level extension* of *c*. We may also say, a clabject has a *class facet* per level of its multi-level extension. That is, a clabject with potency *n* has $n+1$ class facets (including its singleton class facet). In the opposite direction, the clabject is member of $m-n$ clabjects, one class for each level above itself, plus itself as a singleton class. We refer to this as the multi-level classification of *c*.

##### *Example 3*

(Multi-level extension and multi-level classification of a clabject—Figs. 4, 5) Clabject Car has members at level 0, such as MarysCar, members at level 1, such as AcmeS22i, and itself as member at level 2. Clabjects Bike and Accessories may also have members at levels 1 and 0, but they are not (yet) modeled. Clabject Car has itself as class at level 2 and Product as class at level 3. Figure 4 shows an UML diagram of the different class and objects facets of Product, Car, AcmeS22i, and MarysCar. With regard to these different class and object facets, we also say, the object facet of clabject Car (represented by object Car in Fig. 4) is instance of the level-2 class facet of clabject Car (singleton class Car-2) which is a specialization of the level-2 class facet of clabject Product (class Product-2); the object facet of clabject AcmeS22i (object AcmeS22i) is instance of the level-1 class facet of clabject Car (class Car-1); the object facet of clabject MarysCar (object MarysCar) is instance of the level-0 class facet of clabject Car (class Car-0). The instantiation relationship between clabject Car and Product has multiple facets, which are represented in Fig. 3 by three specialization relationships (Car-0, Car-1, and Car-2 are specializations of Product-0, Product-1, and Product-2, respectively).

Sometimes one is interested in the number of instantiation steps that are between a clabject and its class at some higher level, which is the difference of their potencies. Given a clabject *c* at level *m* that has clabject *d* as class at level $m+n$, we say *c* is memberˆn of *d*, or *d* is the classˆn of *c*. Every clabject is its own memberˆ0 and its own classˆ0.

##### *Example 4*

(Memberˆn and classˆn—Figs. 4, 5) MarysCar at level 0 is a memberˆ2 of Car at level 2, which is its classˆ2.

#### Level names

The levels of a clabject hierarchy may be given intuitive names. These *level names* should be read together with the name of the root clabject to get full names for the different levels of the hierarchy.

##### *Example 5*

(Level names—Figs. 4, 5) The levels of the product hierarchy are labeled *Category*, *Model*, and *Individual*. Combining the name of the root clabject, Product, with names of the levels, gives full names for the levels of the hierarchy, namely *Product Category*, *Product Model*, and *Product Individual* (also see Fig. 4).

Combining the name of a clabject with the names of instantiations levels below the clabject gives intuitive names for the clabject’s class facets.

##### *Example 6*

(Names of class facets—Figs. 4, 5) Combining the name of clabject Car with level names gives intuitive names for Car’s class facets. Car’s level 0 class facet is named *Car Individual* and its level-1 class facet is named *Car Model*.

#### Special kinds of clabject hierarchies

A *singleton clabject hierarchy* with a root clabject with potency 0 represents a *self-describing object*. Such a clabject is an object and its own singleton class. In this regard, we say a clabject is its own classˆ0 and its own memberˆ0.

##### *Example 7*

(Singleton clabject hierarchy—Fig. 5) The clabject ACME Corporation with potency 0 represents a self-describing object and holds properties that are relevant for the ACME information system and are only instantiated once for the whole system, such as the *CEO* of the ACME corporation or the products it currently offers. The clabject introduces these properties and instantiates them.

A *two-level clabject hierarchy* typically represents what is traditionally modeled by a single class and its instances. The differences with traditional approaches are that the class itself is also treated as an object with its own identity and description and that an instance may introduce additional properties which it does not share with the other members of the class. Two-level clabject hierarchies typically do not need level names for an intuitive understanding.

##### *Example 8*

(Two-level clabject hierarchy—Fig. 5) The clabject hierarchy rooted in clabject Person is a two-level clabject hierarchy with a class Person and its instances MrWhite, MsBlack, Mary, Peter. The class clabject Person is itself treated as an object with own property DPO (explained later). The individual person clabjects specify the age of the individual persons and, clabject Mary, in its role as a singleton class, adds a property marysSpouse, which it instantiates with Peter.

### Modeling properties with dual potencies

Clabjects are characterized by their properties. We introduce deep properties with dual potencies to generalize the two-level notions of member properties and own properties, to cope with inverse properties, and with clabject hierarchies with different numbers of instantiation levels.

In this section, we discuss different cases of deep property declarations together with flat statements. Deep statements (i.e., statements that do not only describe their source and target but also the members of their source and target) are discussed in Sect. 4.

**Fig. 6:**
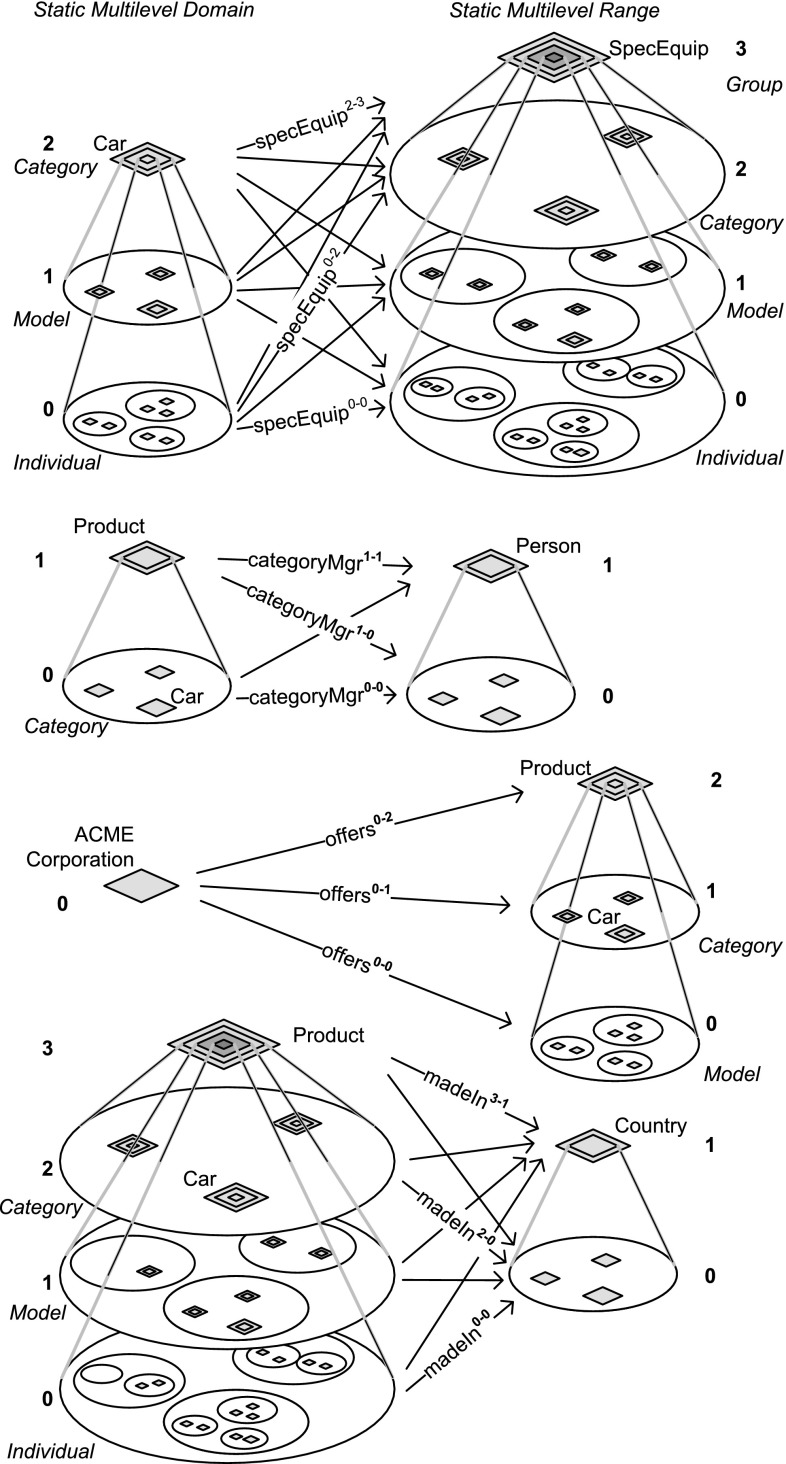
Some examples of dual deep properties, showing their multi-level domain and multi-level range and their multiple simple-property facets in between. Each simple-property facet can be identified by its dual potency which identifies the level of the domain and the level of the range it connects. Typically only a selection of the simple-property facets is instantiated explicitly by statements in a dual deep model

#### Structure of deep properties

In DDM, a *deep property* (or dual deep property or simply *property*) is introduced between a *source* clabject *s* and a *target* clabject *t* and indirectly between a *source hierarchy* and a *target hierarchy*, i.e., the clabject hierarchies of source and target, respectively. As domain and range, it has not only the direct members of these two clabjects; the deep property additionally comes with a source potency *i* and a target potency *j* to separately indicate the number of levels beneath the source clabject that fall in the *multi-level domain* of the property and the number of levels beneath the target clabject that fall in the *multi-level range* of the property.

Source potency *i* and target potency *j* of a property are specified relative to the clabject hierarchy level *k* of the source clabject and the clabject hierarchy level *l* of the target clabject, respectively, with $i \le k$ and $j \le l$. The numbering of the levels of the property’s domain is from bottom (0) to top (*i*), and it contains the source clabject *s* at the top level *i* and its descendants down to its membersˆi at bottom level 0. The multi-level range has the target clabject *t* of the property at its top level *j* and contains the descendants of *t* down to the membersˆj of *t*.

##### *Example 9*

(Multi-level domain and multi-level range—Figs. 5, 6) Clabject Car resides at level 2 of the product hierarchy, but with regard to different properties, it resides at different levels of multi-level domains and ranges of properties (this is illustrated in Fig. 6). It is first at level 2 of the domain of property specEquip. It is at the bottom level 0 of the domain of property categoryMgr. It is at level 1 of the range of property offers, and it is at level 2 of the domain of property madeIn.

A *flat statement* connects a memberˆi of the source clabject of the property with a memberˆj of the target clabject of the property. Flat statements have 0 as source potency and 0 as target potency (i.e., they cannot be further instantiated and are not propagated further down the clabject hierarchies). Higher-level statements (deep statements) are discussed in Sect. 4.

#### Own, member, and memberˆn properties

A deep property that resembles a traditional *member property* is declared with source potency 1 and target potency 1. It is instantiated by flat statements connecting members of the source clabject with members of the target clabject.

##### *Example 10*

(Member property—Fig. 5) Clabject Person as source introduces property age with target clabject Years with source potency 1 and target potency 1. It is instantiated between members of Person and Integer such as by the flat statement MrWhite age 47 Years.

A deep property that resembles a traditional *own property* is declared with source potency 0 and target potency 1. It is not propagated to members of the source clabject but is instead instantiated by the source clabject itself taking a value from the members of the target clabject.

##### *Example 11*

(Own property—Fig. 5) Clabject Person as source introduces property DPO (data protection officer) with source potency 0 and target clabject Person with target potency 1. Clabject Person does not propagate this property to its instances but instantiates it itself with flat value Peter, an instance of Person. In this regard, Person acts as its own class (its 0-class).

A 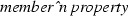 generalizes own properties and member properties to multiple levels. It is a property with source potency *n* and target potency 1 and is propagated down to the membersˆn of the source clabject where it is finally instantiated.

##### *Example 12*

(Memberˆn property—Fig. 5) Clabject Product introduces property owner with source potency 3 and target Person with target potency 1. A flat owner-statement connects clabject MarysCar, a memberˆ3 of Product, with Mary, a memberˆ1 of Person.

#### Flat and deep range

The range of a property is traditionally given by the class of the objects that may be used as values of the property, we also refer to this as a *flat range*. In DDM, this is modeled by a property with target potency 1.

##### *Example 13*

(Flat range—Fig. 5) Clabject Product introduces property owner with target clabject Person and target potency 1.

In DDM, the modeler may additionally specify properties with a deep range. A target potency above 1 indicates a deep range.

##### *Example 14*

(Deep range—Fig. 5) Clabject Car introduces property specEquip with target clabject SpecEquip and target potency 3. The range of specEquip has depth 3 which means that the targets of flat statements on property specEquip are three instantiation levels below clabject SpecEquip.

With dual potencies, the modeler is able to flexibly combine the principles of own/member/memberˆn properties and of flat/deep range.

#### Inverse properties

Every property implicitly introduces an *inverse property*. Inverse properties are used to read and write statements from the opposite directions. For the inverse property, the source clabject and potency become the target clabject and potency, respectively, and vice versa.

Inverse properties are a means to implement bidirectional relationships as required in our database setting. Bidirectional relationships at the conceptual level simplify the definition of the different user views at the external level. This comes with trade-offs at the conceptual level and the internal level. At the conceptual level, inverse properties pose challenges by introducing possible conflicts by concurrent statements on a property and on its inverse property. We tackle these challenges in Sect. 4. Efficient storage and querying of inverse properties have to be dealt with at the internal level by efficient index structures and similar.

##### *Example 15*

(Inverse property—Fig. 5) Property owner has property inv(owner) as inverse property. The latter has Person and 1 as source clabject and potency, and Product and 3 as target clabject and potency. The statement ‘MarysCar owner Mary’ can then be read and written from the opposite direction as ‘Mary inv(owner) MarysCar’ (to-be read as ‘Mary is-owner-of MarysCar’) .

#### Semantic data types

Clabject hierarchies can be used to represent semantic data values, types, and metatypes. Details will be given together with the formalization in F-Logic at the end of this section.

##### *Example 16*

(Semantic data types—Fig. 5) Clabject Car introduces property mileage with semantic data metatype LengthMeasure as target clabject and 2 as target potency. Semantic data types, such as km and mile, are instances of LengthMeasure. Clabject MarysCar has semantic data value 27,000 km, which is an instance of km, as value of property mileage. Note, the clabject hierarchy defining clabjects LengthMeasure, km, and 13,000 km is not depicted in Fig. 5.

### DDM-FL: formalization and implementation of the dual deep modeling approach in F-Logic

In this subsection, we will first give a very brief introduction to Flora-2, a variant of F-Logic, and then introduce the formalization and implementation of the core DDM constructs using the Flora-2 language and system. The resulting formalization and implementation of DDM are referred to as DDM-FL. DDM-FL will be extended in subsequent sections to cover all the different modeling constructs of DDM.

#### Basics of Flora-2

Flora-2^1^ is, first, a language based on F-Logic [25] with metamodeling features based on HiLog [11] and, second, a system implementing this language on top of Prolog system XSB.^2^ Flora-2/F-Logic is related to the Object Constraint Language (OCL) but comes with a more compact syntax and more powerful and flexible metamodeling capabilities. Flora-2 can be regarded as an object-oriented and metamodeling-enabled variant of Datalog. Disregarding any technical and logical details, a Datalog program consists of rules and facts, the rules are executed repeatedly on the facts to derive additional facts until no more facts can be derived. Table 2 gives an informal overview of syntactic elements of Flora-2 used in this paper. For an in-depth introduction to Flora-2, we refer the interested reader to the User’s Manual for Flora-2.^3^

**Table 2:** Flora-2 syntax elements, as used in this paper

*Object identifiers, specialization, and instantiation*	
o:C. C:M.	o is instance of C which is instance of M
v:C. v:D.	v is instance of C and of D
C::D	C is subclass of D
f(a,b):C	Object f(a,b) is instance of C

*Signatures, schema specification*	
$\mathtt{C[p\{i:j\}*=>D]}$	Property or method p at class C with a minimal cardinality i and a maximal cardinality j has range D
C[m(E)*=>D]	Method m at class C with an argument of class E has range D

*Frames and predicates*	
o[p->v]	o has v as value of p (o may also be a class))
o[m(e)->v]	Method m with argument e applied on object o returns v
p(a,b).	A fact of predicate p with arguments a and b

*Syntactic sugar*	
o.p[s->?x]	Dot notation: shorthand for o[p->?y], ?y[s->?x].
o[p->{v,w}]	Set notation, equivalent to o[p->v]. o[p->w].
o[{p,s}->v]	Set notation, equivalent to o[p->v]. o[s->v].

*Rules, queries, and constraints*	
?- ?x:C	Query: Variable ?x is bound to instances of C
a(?x) :- b(?x)	A rule, in first-order logic: $\forall x: b(x) \rightarrow a(x)$
?x:C :- ?x:D and naf ?x:E	Rule with negation as failure: if ?x is instance of D and not instance of E, then it is an instance of C
f(?a):C :- ?a:D	A rule that constructs for every object ?a with class D an object f(?a) with class C
@!{Q(?x)} !- ?x:{C,D}.	Latent query labeled Q with result variable ?x, expressing the integrity constraint that an object ?x should not be instance both of C and D

#### Linguistic metaclasses in DDM-FL

The F-Logic formalization of clabject hierarchies and deep properties is shown in Listing 1. The linguistic metaclasses ClabjectHierarchy, Clabject, and deep Property (see Fig. 3) are represented in F-Logic by classes H, C, and P, respectively. The set of possible potency numbers is represented by class N. In the following, we discuss in detail the definition of the structure of dual deep models in terms of the signatures of these linguistic metaclasses and their semantics in terms of derivation rules as well as global integrity constraints specified as so-called latent queries. The usage of DDM-FL is exemplified in Listing 2, which are discussed at the end of this subsection.

#### Clabject hierarchies in DDM-FL

Clabjects are organized in multi-level clabject hierarchies (see signature of class H at line 1:1, that is line 1 in Listing 1). Every clabject hierarchy has a single root clabject (attribute root at line 1:1) with a root potency (attribute rootPty at line 1:2). The root potency of a hierarchy specifies the number of instantiation levels of the clabject hierarchy. Clabjects at the same instantiation level have the same potency.

The modeler may give an intuitive name to each instantiation level of a clabject hierarchy. Since these names do not affect the formalization of the approach, the naming of instantiation levels is left to the frontend.

A clabject may be instance of one clabject, which acts as its class. In F-Logic, this is represented by the signature of attribute in (see line 1:3) which has minimal cardinality 0 and maximal cardinality 1 and a type constraint to class C.

Every clabject has one potency, which is a nonnegative integer value. In F-Logic, this is represented by the signature of attribute pty of class C (line 1:5) which has cardinality 1:1 and a type constraints to class N which has the set of potential potency numbers as members.
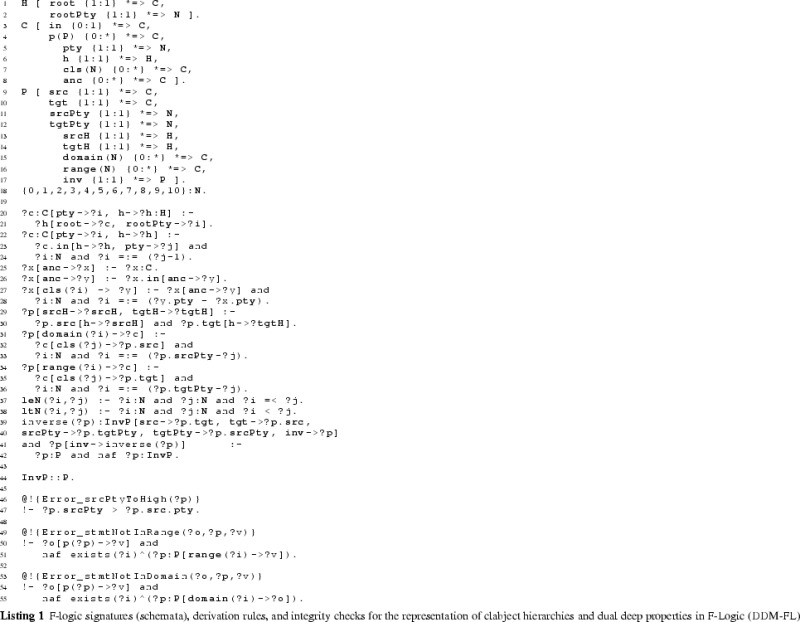
 
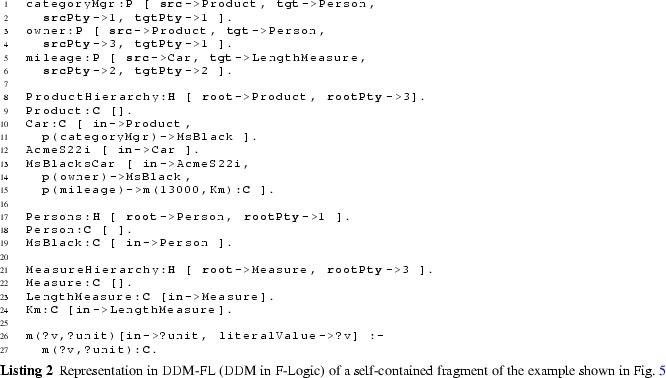


The modeler creates a clabject (see signature of class C at lines 1:3 et seq) either as root of a hierarchy (see above) or as an instance of another clabject which acts as class of the clabject (attribute in at line  1:3).

Derived methods of the clabject metaclass C (see lines 1:5-1:8) are its potency (attribute pty), its hierarchy (attribute h), its classesˆn (attribute cls(N)). Disregarding the number of instantiation steps, its classesˆ* are also referred to as its ancestors (attribute anc). We also say a clabject is a memberˆn of its classˆn, and a clabject is a descendant of its ancestors.

When specifying a hierarchy, the potency of the root clabject is set to the root potency of the hierarchy and the hierarchy attribute of the root clabject is set to the hierarchy (see derivation rule at line 1:20).

A clabject belongs to the same hierarchy as its class. The clabject’s potency is its class’s potency decremented by 1 (see derivation rule at line 1:22).

The ancestor relation is reflexive (line 1:25) and connects a clabject to its class clabjects at the various levels (the derivation rule at line  1:25 propagates the set of ancestors of the class to the instance). The classˆn relation (attribute cls) is the same as the ancestor relation but additionally has the number of instantiation steps as an argument (line 1:27) which is the difference between the potency of the ancestor clabject and the clabject at hand (line 1:28).

The possible potency numbers are explicitly declared as members of class N (line 1:18) to avoid the need of using simple data types in signatures. Their order is represented by binary predicates leN (potency number at the first position is lower than or equal to the potency number at the second position) and ltN (potency number at the first position is lower than the potency number at the second position) (lines 1:37 and  1:38) to avoid numerical comparisons in rules.

#### Deep properties in DDM-FL

A property (class P) is declared between a source clabject (attribute src at line 1:9) and a target clabject (attribute tgt at line 1:10) and is introduced with dual potencies, the source potency (attribute srcPty at line 1:11) and the target potency (attribute tgtPty at line 1:12). Every property has one derived inverse property (attribute inv at line 1:17). By connecting a source clabject to a target clabject, a property also connects a source clabject hierarchy (attribute srcH at line 1:13) with a target clabject hierarchy (attribute tgtH at line 1:14). The source potency of a property must not be higher than the potency of its source clabject (latent query Error_srcPtyToHigh at line 1:47)—due to the rules for inverse properties (see below) this also constrains the target potency not to be higher than the potency of the target clabject. Source clabject hierarchy and target clabject hierarchy of a property may be the same, and also source clabject and target clabject of a property may be the same.

The static multi-level domain of a property (attribute domain(N) at line 1:15) is the multi-level hierarchy of clabjects which may have a statement for that property. The static multi-level domain of a property is always a part of its source clabject hierarchy. The multi-level domain of a property is specified by the property’s source clabject and source potency. A clabject is in the *i*th level of the domain of a property, if it is a memberˆj of the source clabject and *i* is greater equal 0 and is the difference between the source potency of the property and *j* (see derivation rule at lines 1:31-1:33). The static multi-level range of a property (attribute range(N) at line 1:16) is derived analogously (see lines 1:34-1:36).

When the modeler creates a property *p*, the system automatically creates an inverse property inverse( *p* ) (see lines 1:40–1:43). The inverse property is an instance of metaclass InvP which is a specialization of metaclass P (see line 1:45): everything that applies to properties also applies to inverse properties, with this property-creating rule as the only exception (see line 1:43). The source clabject and potency of *p* become the target clabject and potency of its inverse property, and the target clabject and potency become the source clabject and potency of its inverse. Finally, property *p* is the inverse of its inverse property.

A statement links two clabjects, one acting as source and the other as target. In F-Logic, this is represented by method p with the property as an argument and the target clabject as value (line 1:4). The source clabject and the target clabject of a statement must be from the static multi-level domain and range, respectively, of the property. This integrity constraint of the linguistic metamodel is expressed in F-Logic as the latent queries at lines  1:51-1:56. Latent queries in Flora-2 are similar to views in an SQL database, and they are used for expressing integrity constraints of a linguistic metamodel that cannot be expressed as signatures of some class. Integrity constraint violations can be queried by executing the latent queries expressing the integrity constraints, and, in an integrated modeling environment, the results of these queries can be shown to the modeler as warnings and modeling errors.

#### Examples

The usage of DDM-FL is exemplified in Listing 2, which represents parts of the example shown in Fig. 5. Properties categoryMgr (line 2:1), owner (line 2:3), and mileage (line 2:5) are introduced together with their source and target clabjects and potencies. Clabject hierarchy ProductHierarchy (line 2:8) is introduced with one clabject for each of its instantiation levels, exemplifying the representation of instantiation hierarchies and statements in DDM-FL.

Semantic data types and values are organized in a three-level clabject hierarchy MeasureHierarchy (line 2:21), representing semantic data types (or measure units), such as km, and semantic data metatypes, such as LengthMeasure. Semantic data values, such as ‘13000 km’, are represented as clabjects identified by compound terms, such as m(13000 km) (line 2:15). A derivation rule (line 2:26) extracts from the compound term its literal value, e.g., 13000, and the semantic data type, e.g., Km, which is used as class of the clabject.

## Deep statements

In this section, we discuss deep statements (also referred to as higher-level statements) connecting clabjects at arbitrary levels of the multi-level domain with clabjects at arbitrary levels of the multi-level range. We discuss the two facets of properties, namely their schema facet (domain and range) and their instance facet (property values). Property statements at higher levels are, first, regarded as higher-level property values and, second, as refinement of the property’s range, i.e., a restriction of the set of possible values. Concurrent statements for one property at different levels and/or on the property and on the inverse property may introduce conflicts that render some property values inconsistent. We define auxiliary methods in the F-Logic formalization/implementation for querying the schema facet as well as the instance facet of deep properties. This provides the foundation for tools supporting modelers in the flexible refinement and instantiation of multi-level models.

Dual deep modeling generalizes the principle of association end refinement (property redefinition) to multi-level modeling. DDM allows to query the range, the values, and the active range of a property separately for each simple-property facet of the property.

**Fig. 7:**
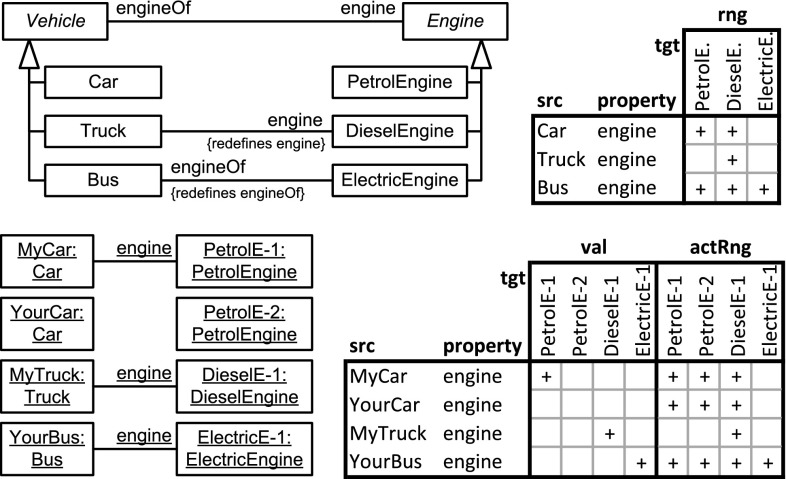
*Top left* An example of association end refinement in the UML using property redefinition showing the interactions between redefinitions of both ends of the association. *Top right* A compact representation of the range of property engine at each concrete subclass of abstract class Vehicle in terms of concrete subclasses of Engine (indicated by ‘$+$’), and vice versa, taking into account the redefinitions of property engine and its inverse engineOf. For example, class Car has class PetrolEngine and class DieselEngine as range of engine, and, in the other direction, PetrolEngine has Car and Bus in the range of property engineOf. *Bottom left* An UML object diagram in line with the above class diagram. *Bottom right* A compact representation of the property values (or links), and of the active range (possible values/links), of the objects in the object diagram on the left. For example, object MyCar has PetrolE-1 as value of property engine, and, vice versa, PetrolE-1 has MyCar as value of property engineOf. Based on the objects modeled in the object diagram and the class diagram, object MyCar has objects PetrolE-1, PetrolE-2, and DieselE-1 as active range of property engine

### Background from two-level modeling: property redefinition, value, range, and active range

In two-level modeling with class generalization, property *refinement* refers to the case when a subclass specifies a more specific class as range of a property then its superclass. In the UML, property refinement is possible through the redefinition of association ends [13] or attributes. The UML class diagram at the top left of Fig. 7 shows an example in which both ends of a binary association are redefined by associations at subclasses. These redefinitions interact in a way that the range of a property is affected by redefinitions of its inverse property.

The *range* of a property *p* at a class *c* is the set of classes from which a member of *c* may take its values for property *p*, taking into account property definitions and redefinitions. If a class *d* is in the range of property *p* at class *c*, then *c* is in the range of the inverse of *p* at *d*. The ranges of a property at different classes that have this property can be concisely represented by a table (see top right of Fig. 7).

At the instance level, the set of objects that may act as value of property *p* for an object *o* in accordance with the schema is referred to as the *active range* of *p* at *o*. If an object *x* is a value, or is in the active range, respectively, of property *p* of object *o*, then object *o* is a value, or is in the active range, respectively, of the inverse of property *p* of object *x*. In an object diagram that is consistent with its schema, property values are taken from the active range of the property. Values and active ranges of a property (and its inverse property) at the different objects can be concisely represented by a table (bottom right of Fig. 7).

Dual deep modeling generalizes property redefinitions, as well as these bidirectional notions of property value, active range, and range, to multi-level modeling.

### Modeling with deep statements

A *deep statement* (or simply *statement*) on property *p* connects a clabject from *p*’s multi-level domain with a clabject from *p*’s multi-level range, the former in the role of source or owner of the statement and the latter in the role of target or value.

Source potency and target potency of a statement are not asserted but derived. The source potency of a statement is calculated from the source potency of the property declaration minus the number of instantiation steps between the source clabject of the statement and the source clabject of the property declaration. The target potency is calculated analogously.

A deep property has multiple *simple-property facets* (also referred to as *levels of a deep property*), one for each combination of source and target potencies (see Fig. 6). A statement on property *p* with derived source potency *i* and derived target potency *j* instantiates *p*’s level-*i*-*j* simple-property facet.

A deep statement has multiple roles with regard to the *instance facet*  and the *schema facet*  of these simple-property facets. First, a deep statement represents a property *value* (instance facet) for the simple-property facet it instantiates. Second, deep statements represent range refinements (schema facet) for all lower-level simple-property facets: A statement for a property between a source clabject and a target clabject and a derived source potency *i* and a derived target potency *j* affects the set of allowed values for the property at membersˆ(0...i) of the source clabject, not only concerning values at target potency *j* but also at lower target potencies. In this regard, the derived source and target potencies of a statement indicate how many instantiation levels of the source and of the target are affected by the statement.

**Fig. 8:**
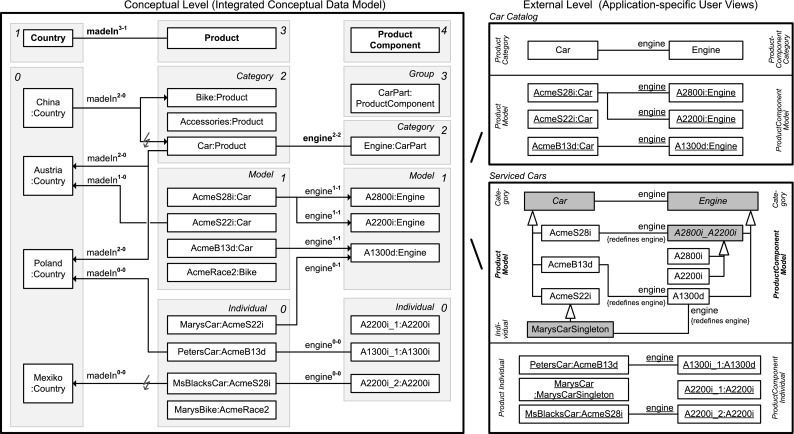
A fragment of the ACME enterprise database with deep statements including statements on inverse properties. Introduced conflicts (depicted by a bolt symbol) are detected and resolved. The user views (*right*) represent two-level excerpts from the integrated multi-level model

#### Two-level user views

Different *two-level user views* may be defined on top of a multi-level model and emphasize different levels of clabject hierarchies and properties. A two-level user view projects the multi-level model to a traditional two-level model with schema and instance level.

Two-level user views not only make multi-level models accessible for two-level front-end applications; they also allow to illustrate the semantics of DDM in the more familiar terms of the UML. A two-level user view is defined on top of a multi-level conceptual model by a set of class facets of clabjects (each specified by a clabject and a potency) and a set of simple-property facets of deep properties (each specified by a deep property and a source potency and a target potency).

The schema of a two-level view consists of classes and properties (attributes and associations) that reflect the selected class facets and simple-property facets and their specializations and redefinitions which are taken from different levels of the multi-level model.

The instance level of a two-level view consists of the members of the selected class facet and the values (attribute values or links) of the selected simple-property facets. More details on the correspondence between two-level views and multi-level model are given in the following. A full formalization, however, of two-level user views and their derivation from and interactions with the multi-level enterprise database is beyond the scope of this paper.

##### *Example 17*

(Two-level user views—Fig. 8) Application-specific user views such as *Car Catalog* and *Serviced Cars* (depicted by UML class and object diagrams at the right of Fig. 8) at the external level of the ACME enterprise database are defined as a collection of single-level facets of clabject hierarchies and simple-property facets of properties and are derived from the multi-level database (at the left of Fig. 8). User view *Car Catalog* in the top right part of Fig. 8 is defined by the level-1 class facet of clabject Car, the level-1 class facet of clabject Engine, and the level-1-1 simple-property facet of property engine. The classes of user view *Car Catalog* are taken from level 2 of the product hierachy (labeled *Product Category*) and level 2 of the product component hierarchy (labeled *Product Component Category*); the objects of user view *Car Catalog* are taken from level 1 of the product hierachy (labeled *Product Model*) and level 1 of the product component hierarchy (labeled *Product Component Model*). User view *Serviced Cars* in the bottom right part of Fig. 8 is defined by the level-0 class facets of clabjects Car and Engine and the level 0–0 simple-property facet of property engine. The classes in the class diagram of user view *Serviced Cars* represent class facets of different levels of clabjects at different levels of the multi-level model (namely levels *Product Model*, *Product Individual* and *Product Category* from the product hierarchy; and levels *Product Component Model*, *Product Component Category* from the product component hierarchy). Further explanations are given in the following examples.

A deep property may be instantiated at all its levels, but typically only a smaller set of simple-property facets is actually instantiated. It is also possible that some simple-property facets are frequently instantiated and others are only instantiated rarely. Typically, user views are defined only at the levels that are instantiated frequently. An *intermediate statement* is a statement at a level that is instantiated infrequently and is thus represented in user views only at the schema level of lower-level user views.

##### *Example 18*

(Intermediate statements—Fig. 8) The particular engine of clabject MarysCar is not known, but it is known that the engine is an instance of engine model A1300d. This is represented in the multi-level model by statement ‘MarysCar engine A1300d’ with source potency 0 and target potency 1. At the instance level, this statement is a value of the level 0–1 simple-property facet of engine, which is, in our example, not part of a user view. At the schema level of user view *Serviced Cars*, the statement is reflected by a singleton class MarysCarSingleton that refines the range of (the level 0–0 simple-property facet of) property engine to class A1300d.

#### Missing statements, single statements, and statement ensembles

For a given target potency, a clabject in the multi-level domain of the property may have no statement, a single statement, or multiple statements (called a *statement ensemble* ) instantiating the property. We now discuss these three basic cases and their interpretation regarding the schema facet (range) and instance facet (value) of the property.

We talk of a *single statement* when a source clabject has only one statement for a property and a given target potency. At the instance level, a single statement is interpreted by a property value connecting the object facet of the source clabject with the object facet of the target clabject. At the schema level, a single statement is interpreted by property range refinements connecting the class facets of the source clabject with the class facets of the target clabject, one refinement for each simple-property facet beneath the single statement.

##### *Example 19*

(Instance and schema facet of a single statement—Fig. 8) Clabject AcmeB13d at level 1 of the product hierarchy in the multi-level model shown in the left part of Fig. 8 is source of a single statement with target potency 1 for property engine with clabject A1300d as target. At the instance level, this is a property value (or link) connecting object AcmeB13d with object A1300d. This is represented, for example, at the instance level of user view *Car Catalog* (top right of Fig. 8) by a link between objects AcmeB13d and A1300d. At the schema level of all lower-level simple-property facets, the statement refines the range for the class facets of AcmeB13d to the class facets of A1300d. This is represented, for example, in the schema part of user view *Serviced Cars* (bottom right of Fig. 8), by an association between class AcmeB13d and class A1300d.

A *statement ensemble* is a *non-empty set* of statements with the same source clabject, the same property, and the same target potency. A statement ensemble may consist of a single statement. At the instance level, every statement represents a property value. At the schema level, a statement ensemble is interpreted by the union of targets, that is, at this range level and at all lower levels the range is refined to the union of the respective class facets of the target clabjects. The assumption behind this is that a statement ensemble fully describes the source clabject with regard to the given property and target potency, or, in other words, if a clabject comes with data for a target level of a property, then we assume these data to be complete. In UML, such a multi-target range refinement is expressed by a redefinition of the association end with an additional ‘union class’ as type—the introduction of such additional union classes may lead to a combinatorial explosion which is avoided by statement ensembles.

##### *Example 20*

(Instance and schema facet of a statement ensemble—Fig. 8) Clabject AcmeS28i in the left part of Fig. 8 is source of two statements with target potency 1 for property engine with clabjects A2800i and A2200i as targets. These two statements constitute a statement ensemble. At the instance level, this is reflected by two property values connecting object AcmeS28i with objects A2800i and A2200i (see object diagram of user view Car Catalog). At the schema level of this and all lower-level simple-property facets, the statements refine the range for the class facets of AcmeS28i to the union of the class facets of A2800i and A2200i. This is represented, for example, at the schema level of user view *Serviced Cars* by an association that redefines property engine at class AcmeS28i to class A2800i_A2200i, which represents the union of classes A2800i and A2200i.

When a clabject in the domain of a property does not own a statement for the property, we talk of a *missing statement*. Similar to NULL values in relational databases, missing statements have various reasons. Disregarding other more specific cases, we interpret a missing statement simply as ‘no information’ with no effects on the schema and instance level.

##### *Example 21*

(Instance and schema facet of missing statements—Fig. 8) Clabject AcmeS22i has no statements for property engine. Consequently, the object AcmeS22i (shown with user view *Car Catalog*) has no property values and the class AcmeS22i (shown with user view *Serviced Cars*) does not redefine property engine.

#### Deep statements and inverse properties

As introduced before, every property comes with an inverse property. While the direction of a statement is of no interest at the bottom level (with 0 as source and target potency) of a property, it is of interest at higher levels, where statements also refine the range of the property at lower levels. *Statements on a property’s inverse* refine the domain of a property with regard to a clabject in the multi-level range of the property. The interplay between refinement of a property and refinement of the property’s inverse may lead to inconsistencies. Adequate tool support will catch such inconsistencies according to the semantics defined by our F-Logic formalization.

##### *Example 22*

(Statements on a property’s inverse—Fig. 8) The links from Car to Austria and Poland express statements made with clabject Car that cars are only made in Austria and Poland. The statement from China to Car on madeIn’s inverse as well as the statement from MsBlacksCar to Mexiko are inconsistent with the above. The DDM system detects these inconsistencies and gives warnings to the modeler. Further, when querying the range or the values of property madeIn and its inverse, these inconsistent statements are disregarded.

#### Active range

Higher-level statements act as range for lower-level statements, and this is reflected in the *active range* of properties. The active range of a property is the set of all possible links that can be inserted into the database without violating a range refinement on the property or the inverse property, introduced by a higher-level statement.

##### *Example 23*

**Fig. 9:**
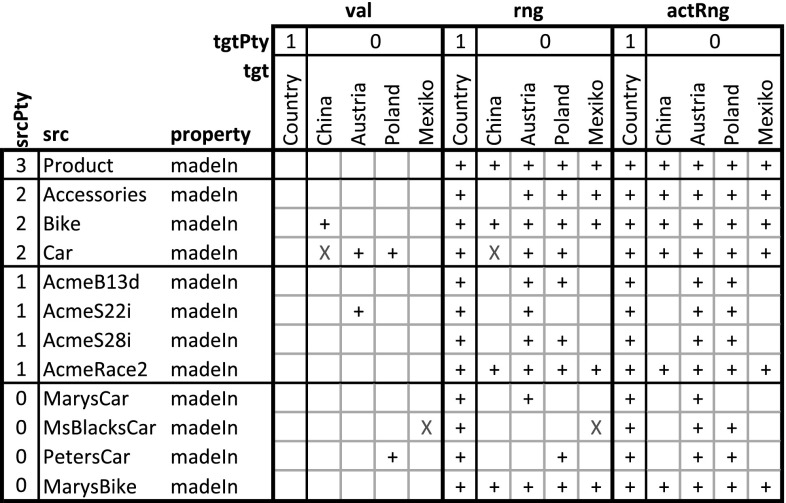
Compact representation of the results of querying the multi-level model in Listing 4 for the values (val), the range (rng), and the active range (actRng) of property madeIn. Since value, range, and active range are bidirectional, this matrix also gives values, range, and active range for property madeIn.inv. ‘$+$’ indicates the existence of a property instance (either val, rng or actRng), ‘X’ indicates an inconsistent statement (i.e., one that was not in the active range) that has been discarded from the result


(Active range—Fig. 8) The active range of property madeIn at clabject AcmeS22i consists of clabjects Austria and Poland, reflecting the higher-level statements made at clabject Car. The statement ‘AcmeS22i madeIn Austria’ is in this active range and sets Austria as range and value of property madeIn at clabject AcmeS22i. See Fig. 9 for the active range of property madeIn at the different clabjects in the product hierarchy.

#### Flexibility

Statements in DDM support a very flexible refinement of the property’s range.

- Source and target instantiation levels may be freely skipped.
- If a clabject inherits a refined range for target potency *j*, it may freely make additional statements at target potency *j* or above or beneath.
- Range refinements are single-valued (a single statement) or multi-valued (a statement ensemble).
- A range refinement for target potency *j* also affects the range and active range for all lower target potencies (the range at a lower level only contains descendants of the clabjects specified for target potency *j*). The derivation of the range and active range has to consider these different sources of range refinements.
- Clabject hierarchies may be only partially specified; due to incomplete clabject hierarchies, lower levels of the range of a property may be empty, and this should not affect higher levels of the range or active range.

### Extending DDM-FL with deep statements

In this section, the F-Logic formalization of DDM is extended (see Listing 3) for dealing with higher-level statements and for querying property values, range, and active range derived from statements, taking into account also statements on inverse properties. This should provide the foundation for frontend tools (like code completion, and drop down lists) that support modelers in the stepwise refinement and instantiation of multi-level models. The usage of the language constructs is exemplified in Listing 4 which are discussed at the end of this subsection.

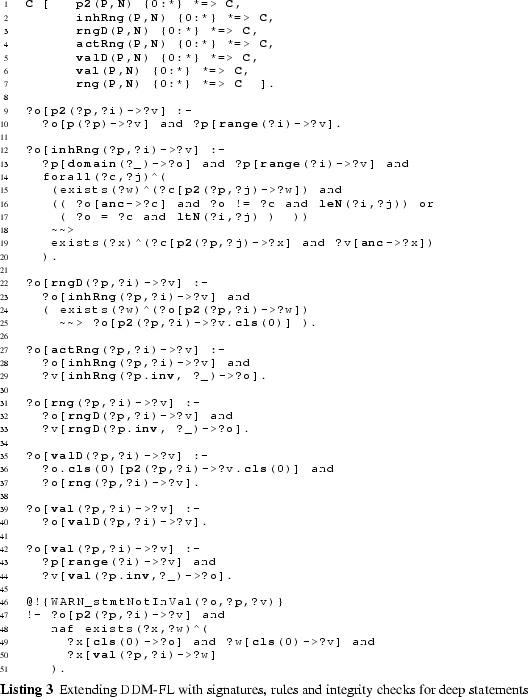


#### Additional methods

The additional methods for querying the values (see signature of method val at line 3:6), the range (method rng at line 3:7), and the active range (method actRng at line 3:4) of a property are defined as methods of class C with the property (a member of P) and the target level (a member of N) as arguments. To this end, we define auxiliary methods for querying statements together with their target potency (method p2 at line 3:1), for querying the inherited unidirectional range (method inhRng at line 3:2), the unidirectional range (method rngD at line 3:3), and the consistent unidirectional values (method valD at line 3:5).

Having the target potency of statements as additional argument helps identify statement ensembles. The rule at line 3:9 derives the target potency of a statement from the level in the multi-level range at which the target clabject resides.

#### Deriving a property’s range

The next and most intricate step is to calculate the unidirectional inherited range (rule at lines 3:12-3:20), which is basically the intersection of all range refinements introduced by higher-level statements. A target clabject *v* is in the inherited range of property *p* at target level *i* for source clabject *o*, if *o* is in the static domain of *p*, and *v* is at level *i* of the static range of *p* and for all superordinate clabjects *c* and target potencies *j* (superordinate here means that *c* is an ancestor of *o* and *i* is below or equal to *j* and either $o \ne c$ or *i* is below *j*) with a statement ensemble for property *p*, target clabject *v* is ancestor of a target clabject *x* of this statement ensemble.

The unidirectional range (rule at lines 3:22-3:25) of source clabject *o*, property *p* and target potency *i* also considers ‘coordinate’ statements. A coordinate statement here is a statement made at source clabject *o*, with property *p* and target potency *i*. If such coordinate statements exist, the intersection between inherited range and coordinate statements gives the unidirectional range, otherwise the inherited range becomes the unidirectional range. This means that a clabject can refine its own range.

The active range (rule at lines 3:27-3:29) is the bidirectional variant of the unidirectional inherited range. It is given by the intersection of the inherited range of the property (line 3:28) and the inverse of the inherited range of the inverse property (line 3:29). This means that the active range gives the set of possible links which already exist in or may be added to the multi-level model without violating any range refinements introduced by superordinate statements.

The formalization and implementation in F-Logic provide for very flexible querying of such derived schema information. For example, an integrated modeling environment may support the modeler by, given two clabjects, provide all properties which can be used to consistently link the two clabjects as source and target of a property.

The range (rule at lines 3:31-3:33) is the bidirectional variant of the unidirectional range. It is given by the unidirectional range of the property (line 3:32) and the inverse of the unidirectional range of the inverse property (line 3:33). The set of tuples in the range of a property can be understood as a set of associations that all derive from one most-general association (which is represented by the property and its inverse) and thus constitute the schema facet of the property.

#### Deriving property values

The consistent unidirectional values (rule at lines 3:35-3:37) of a property *p* at clabject *o* for target potency *i* are given by statements that are consistent with the bidirectional range of the property. The rule also takes into account clabject generalization (see line 3:36), we come back to this in the next section.

The property values (rules at lines 3:39-3:34) are given by the union of the consistent unidirectional values of property *p* and its inverse. Every property value has an inverse property value, which together constitute a link. The set of property values (together with their inverse property values) at the different source and target levels constitutes the multi-level extension (instance facet) of the multi-level association expressed by the property and its inverse property.

#### Covariant refinement

The definitions of the schema facet (range) and instance facet (value) introduced in this section follow a covariant refinement approach. Range and value are derived—basically—by the intersection of all relevant statement ensembles. This can lead to situations where a statement made by the modeler is fully discarded from value and range. Obviously, the modeler should be warned in such situations, so she can decide herself if the conflict resolution applied by DDM-FL by default is adequate or if she wants to resolve the conflict herself. This is the purpose of latent query WARN_stmtNotInVal at line 3:46: For every statement that is not reflected as a property value, the system gives a warning to the modeler (the definition also takes into account clabject generalization, we come back to this in the next section).
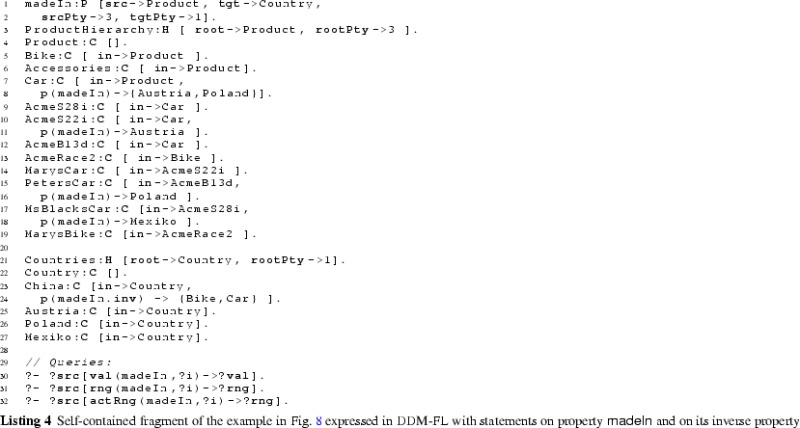


#### Examples

The usage of DDM-FL for expressing deep statements in both directions of a property is exemplified in Listing 4, which represents property madeIn (line 4:1) together with clabject hierarchies Countries (line 4:22) and ProductHierarchy (line 4:4) from the example shown in Fig. 5. Property madeIn is introduced between Product and Country with source potency 3 and target potency 1. Statements at different levels give information about the country of origin of different products. All ACME cars are made in Austria or Poland (line 4:9). AcmeS22i cars are made in Austria (line 4:12). PetersCar was made in Poland (line 4:17) and MsBlacksCar in Mexiko (line 4:19). In the other direction, using the inverse of property madeIn, it is specified with clabject China that bikes and cars are made in China (line 4:25). The latter is inconsistent with the statements made with clabject Car that cars are produced in Austria and Poland. DDM-FL detects this inconsistency and gives a warning to the modeler. The modeler can repair this inconsistency by deleting statement ‘China madeIn.inv Car’ or by adding statement ‘Car madeIn China’. As a default, when querying the range and values of a property, inconsistent links are discarded from the result.

Figure 9 shows the results of queries on the value (see line 4:31), on the range (line 4:32), and on the active range (line 4:33) of property madeIn.

## Clabject generalization in dual deep modeling

Generalization is an ubiquitous construct for abstraction in object-oriented modeling. In this section, we extend DDM with clabject generalization at all levels of identity. When modeling at multiple levels of identity, the challenge with clabject generalization is to maintain, at each modeling level, a clear principle of identity (in order to be able to count the number of clabjects at some level of identity that act as value or range of some property). When generalizing two clabjects to a *super-clabject*, then the super-clabject should not be counted as an individual at the same level of identity as its sub-clabjects. We cope with this ontological problem by making a hard distinction between abstract and concrete clabjects and consider all super-clabjects as abstract. An abstract super-clabject does not come with its own identity. Its main purpose is to act as handle for the set of concrete clabjects it generalizes and factor out their common property values and ranges. Abstract super-clabjects help reduce the size of a multi-level model by reducing the number of statements and thus improve the manageability and understandability of multi-level models. Statements with abstract clabjects are interpreted by their effects on concrete clabjects in terms of concrete property values and ranges. An abstract clabject may further be used to introduce a new property simultaneously for a set of clabjects as source and/or target.

### Background: object and class generalization in two-level models

A set of classes (in the role of subclasses) may be generalized to a class (in the role of the superclass). We also say, the subclasses are specializations of the superclass. A set of superclasses may be further generalized to another superclass, which is then an indirect generalization of the (direct and indirect) subclasses of its subclasses. Generalization may also be applied to objects [27].

Generalization has many facets

- with regard to the extensional facet of a class, generalization is interpreted as subsetting: The extension of the subclass is a subset of the extension of the superclass. We refer to this facet of generalization as *set generalization*.
- with regard to the structural facet of a class, the common properties of a set of classes (subclasses) are moved from the subclasses to the superclass. (we refer to this as *structural generalization*).
- with regard to own property values of objects, common property values of a set of objects are moved to an abstract super-object. We refer to this as *value generalization*.

The distinction between abstract and concrete class is described as: ‘A class that has the ability to create instances is referred to as *instantiable* or *concrete*, otherwise it is called *abstract*.’ [19] The distinction between *abstract objects* and *concrete objects* is heavily discussed in Philosophy [41], and there are many different ways to explain it. We follow ‘the way of abstraction’ as mentioned by Kühne [27] as: ‘An abstract object represents all instances that are considered to be equivalent to each other for a certain purpose [...] An abstract object captures what is universal about a set of instances but resides at the same logical level as the instances.’

In the literature, it has been discussed whether only abstract classes may be specialized. Hürsch [19] postulated the *abstract superclass rule*, stating that all superclasses are abstract in that they have no direct instances, and discussed its advantages and drawbacks. Obeying this rule improves the clarity of object-oriented models, especially when the extension (set of instances) of classes is of interest, with the trade-off of additional classes to be modeled and maintained.

Generalization of objects is used for value generalization, and, in the case of self-describing objects also for structural generalization. In our understanding, an *abstract object* does not represent a proper domain object, it rather only provides a handle to define commonalities of concrete domain objects. The property values of an abstract object do not describe the abstract object itself but rather its concrete specializations. When counting the number of objects in the extension of a class, only concrete objects are counted. For example, when counting the number of persons represented in a database, only the concrete person objects are counted, factoring out of commonalities between persons into abstract person objects does not affect the number of persons represented in the database. We assume that super-objects are abstract.

*Single generalization* is when a class or object has at most one *direct generalization*, it may still have multiple indirect generalizations. *Multiple generalization* is when a class or object has more than one direct generalizations and inherits properties, range refinements, and/or property values from these multiple generalizations. With regard to the different facets of generalization, multiple generalization has the following facets:

- The extension of the subclass is subset of the extension of every superclass, or, formulated differently, the extension of the subclass is subset of the intersection of the extensions of its superclasses.
- A subclass inherits properties from multiple superclasses. The set of properties of a subclass is the union of the sets of properties of its superclasses
- With regard to value generalization, a subclass inherits values from multiple superclasses. The set of inherited property values is the intersection of the property values of its superclasses.

Multiple generalization potentially introduces conflicts, especially with regard to property range refinements (discussed in Sect. 4), conflicting range refinements in superclasses may lead to properties that have an empty range (cannot be instantiated with regard to the range refinements). Furthermore, an object or class may inherit multiple property values for the same property. This may be in conflict with cardinality constraints (discussed in Sect. 7). The possible problems associated with multiple generalization strengthen the need for proper modeling tools with basic reasoning services that help detect and resolve such conflicts.

### Clabject generalization

We first provide a high-level picture of extending DDM with generalization, discussing the role of super-clabjects in multi-level clabject hierarchies. We clarify the distinction between abstract and concrete clabject. We adapt the *abstract superclass rule* to the setting of multi-level modeling with abstract and concrete clabjects.

**Fig. 10:**
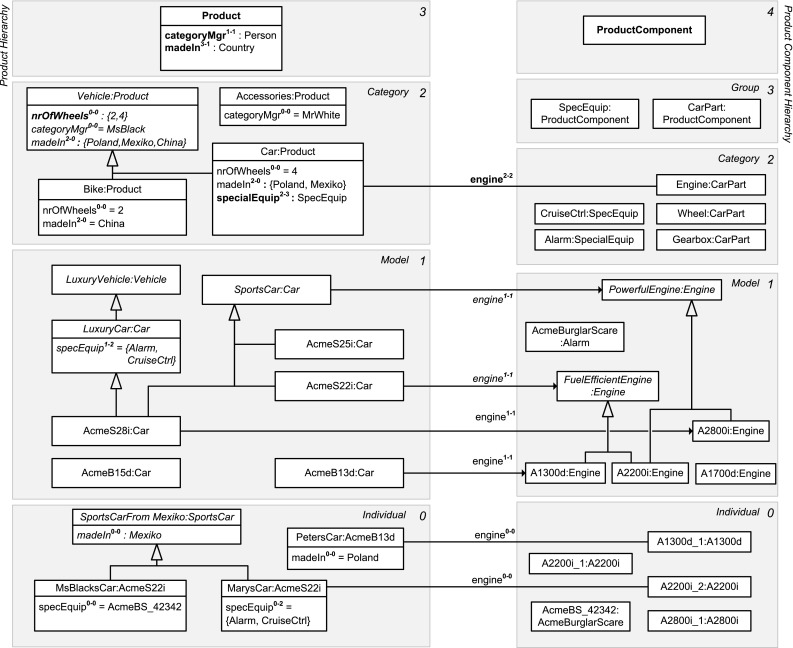
Extending the ACME product knowledge base with abstract super-clabjects and multiple generalization. Abstract clabjects as well as abstract statements are shown in italics

#### The abstract super-clabject rule

In DDM, a clabject is either concrete or abstract. A *concrete clabject* combines concrete class and concrete object (see above). An *abstract clabject* combines abstract class and abstract object (see above), that is, an abstract clabject only has an abstract object facet, meaning that the property values of an abstract clabject do not describe the abstract clabject itself but its concrete specializations. Technically, it means that property values of an abstract clabject are always propagated to its specializations.

We argue that obeying the abstract superclass rule (see above) in multi-level modeling is beneficial because of the increased clarity and helps to maintain a principle of identity for each level of a clabject hierarchy, which is in turn a prerequisite for cardinality constraints with a clear semantics. That is why in the Dual Deep Instantiation (DDI) approach [36], a precursor of DDM, only concrete clabjects could be instantiated. In [37], we relaxed this restriction as follows:

*Abstract Super-clabject Rule:* All super-clabjects are abstract in that they have no direct concrete instances (but they may have abstract instances).

The DDM approach obeys the abstract super-clabject rule.

#### Kinds of instantiation relationships

The meaning of the allowed kinds of instantiation relationships depends on the abstract/concreteness of the related clabjects. An instantiation relationship between a clabject *x* in the role of the instance and a clabject *c* in the role of the class can be classified as *immediate concrete*, *shared concrete*, and *shared abstract*.

An *immediate concrete instantiation* relationship is between a concrete clabject in the instance role and a concrete clabject in the class role.

##### *Example 24*

(Immediate concrete instantiation—Fig. 10) Clabject Accessories has class Product. This represents an immediate concrete instantiation relationship, meaning that Accessories is an instance of Product, or, more exactly, that the object facet of Accessories is an instance of the class facet of Product for level 2. Taking into account the intuitive names of instantiation levels, one can say, *Accessories* is an instance of class *Product Category*.

A *shared concrete instantiation* relationship is between an abstract clabject *x* in the instance role and a concrete class *c* in the class role. It means that all concrete specializations of *x* are instances of a class facet of *c*.

##### *Example 25*

(Shared concrete instantiation—Fig. 10) Abstract clabject Vehicle has concrete clabject Product as class. This represents a shared concrete instantiation relationship and means that all concrete specializations of Vehicle, such as Bike and Car, are instances of Product.

A *shared abstract instantiation* relationship is between an abstract clabject *x* in the instance role and an abstract clabject *c* in the class role. It means that each concrete specialization of *x* is instance of some concrete specialization of *c*.

##### *Example 26*

(Shared abstract instantiation—Fig. 10) Abstract clabject LuxuryVehicle has abstract clabject Vehicle as class. This represents a shared abstract instantiation relationship, meaning that all concrete specializations of LuxuryVehicle are instances of a concrete specialization of Vehicle, e.g., AcmeS28i is an instance of Car.

An *immediate abstract instantiation* (a concrete clabject with an abstract clabject as direct class) is not allowed under the abstract super-clabject rule.

#### Kinds of statements

A *shared* or *abstract* *statement* is a statement with an abstract clabject as source or target, respectively. Abstract statements are interpreted with regard to the *concrete statements* (i.e., statements between concrete clabjects) they imply. In this regard, there are four different cases of statements depending on the abstractness/concreteness of the connected clabjects:

A *concrete statement* is a statement relating a concrete clabject as source and a concrete clabject as target.

##### *Example 27*

(Concrete statement—Fig. 10) Concrete clabject AcmeS28i has concrete clabject A2800i as target of property engine.

A *shared concrete statement* is a statement relating an abstract source with a concrete target. The concrete target is propagated to the concrete specializations of the abstract source. With regard to a fixed set of concrete specializations of the source, the shared statement is equivalent to multiple concrete statements, one for each concrete specialization of the source.

##### *Example 28*

(Shared concrete statement—Fig. 10) Abstract clabject Vehicle has concrete clabject MsBlack as target of property categoryMgr, meaning that product categories Car and Bike have MsBlack as category manager. More technically, the shared statement implies concrete instances of property categoryMgr between concrete clabjects Bike and Car as source clabjects and MsBlack as target clabject.

An *abstract statement* is a statement relating a concrete source *o* with an abstract target *v*. An abstract statement restricts the range to descendants of *v*. At the instance level, an abstract statement is interpreted by a set of property values, one for each concrete descendant of *v*.

##### *Example 29*

(Abstract statement—Fig. 10) Concrete clabject AcmeS22i has abstract clabject FuelEfficientEngine as target of property engine. With regard to the given concrete specialization of FuelEfficientEngine, this can be interpreted as two concrete statements with A1300d and A2200i as targets.

A *shared abstract statements* is between an abstract source and an abstract target. It is propagated as an abstract statement to the concrete specializations of the abstract source clabject.

##### *Example 30*

(Shared abstract statement—Fig. 10) Abstract clabject SportsCar has abstract clabject PowerfulEngine as target of property engine, meaning that every SportsCar model, namely AcmeS22i and AcmeS28i, take its engine from the set of powerful engine models, namely A2200i and A2800i.

#### Resolving multiple inheritance

Multiple generalization may lead to situations where a clabject *c* inherits different property targets for the same property. The derived range of a property *p* at *c* is the set of clabjects that fulfill all the different range refinements introduced by *c* or one of its ancestors. If this set is empty, the property cannot be instantiated. Such a situation may be intended by the modeler or may also be the result of over-constraining the model. Mandatory range constraints (to-be introduced in Sect. 7) provide a flexible means to monitor such situations.

##### *Example 31*

(Resolution of multiple inheritance—Fig. 10) Abstract clabject SportsCar asserts abstract clabject PowerfulEngine (representing concrete clabjects A2200i and A2800i) as target of property engine. Its specialization AcmeS22i inherits these targets and asserts itself FuelEfficientEngine (representing concrete clabjects A1300d and A2200i) as target. The active range of AcmeS22i for property engine at level *model* is thus A2200i, the only engine model that is both fuel efficient and powerful.

### Extending DDM-FL for clabject generalization

In this section, DDM-FL is extended with signatures, rules, and integrity checks for clabject generalizations. Some of the rules extend attributes, namely h, pty, anc, and cls, introduced in Listing 1 in Sect. 3.4. We also revisit some of the auxiliary methods and consistency checks introduced in Listing 3 in Sect. 4.3 The usage of clabject generalization in DDM-FL is exemplified in Listing 6 and discussed at the end of this section.

A clabject may have multiple clabjects as generalizations (denoted by method sp for ‘specialization-of’ at line 5:1). Clabjects may be specified as abstract (line 5:2) with all other clabjects considered as concrete (lines 5:3, 5:7). Concrete clabjects must not be specialized, i.e., must not act as generalizations (line 5:10).

A clabject inherits from its generalizations the potency as well as the hierarchy it belongs to (line 5:5). This expresses, together with the cardinality (1:1) of pty and h (see lines 1:5 and 1:6), that the clabject and all its generalizations belong to the same level of the same hierarchy. We assume that a clabject that has neither a class nor a generalization is the root of a clabject hierarchy.

Every clabject is instance of at most one concrete clabject at the next higher level (line 5:13), and every concrete clabject is instance of a concrete clabject at the next higher instantiation level if there is a next higher instantiation level in that hierarchy (line 5:15).
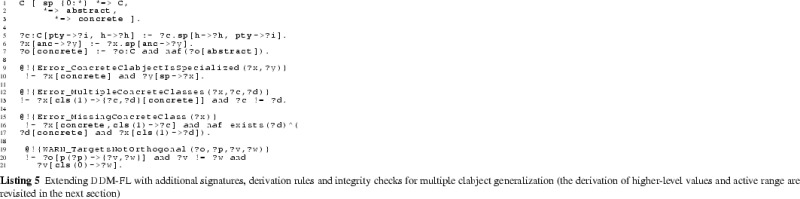
 
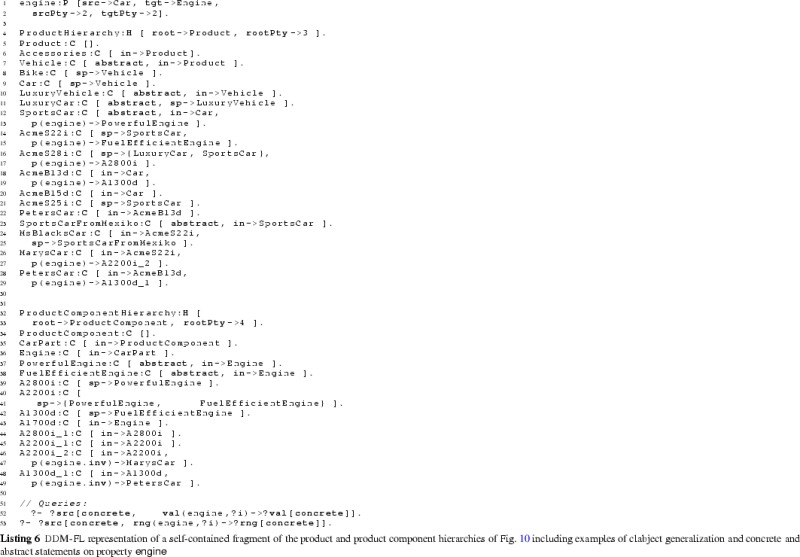


A clabject may have multiple ancestors at the same level (that is, with the same number of instantiation steps between them). The anc method previously introduced (see line 1:8) is extended in order to also consider generalizations as ancestors (line 5:6). This also affects method cls (previously introduced, see line 1:7). The set of super-clabjects, i.e., direct and indirect generalizations, of a clabject including the clabject itself is also referred to as the classesˆ0 of the clabject.

The rule for deriving the unidirectional value of a property (previously introduced, see line 3:36) also takes into account statements between abstract clabjects (classesˆ0) and propagates them to their specializations, formalizing the notions of shared concrete statement and shared abstract statement. Note, the value facet of a statement is shared by the specializations of source and target clabjects but not by their instantiations (at lower modeling levels). Sharing of values across modeling levels is subject to ongoing and future work.

A statement of property *p* connecting a source clabject *o* with a target clabject *v* that is not in the range of *p* typically reflects an unintended modeling situation. When the source or target of such a statement is an abstract clabject, then such a situation may be intended by the modeler as long as there is a specialization of the abstract clabject that gives a consistent property value. The previously introduced latent query WARN_stmtNotInVal (see line 3:44) covers this situation and issues a warning only in situations that are not in line with the following assumptions about the intentions of a modeler: If a statement of property *p* connects a (possible abstract) source clabject *o* with a (possible abstract) target clabject *v*, then *v* may not be in the range of property *p* of *o* as long as there is a clabject *x* (which is *o* or a specialization of *o*) that has a clabject *w* (which is *v* or a specialization of *v*) in the range of property *p* which makes *w* a consistent value of property *p* of clabject *x*. An example of such intended modeling situation is the statement ‘AcmeS22i engine FuelEfficientEngine’ of Fig. 10 which is not in the range of property engine due to statement ‘SportsCar engine PowerfulEngine’ but both statements have an overlap, with ‘AcmeS22i engine A2200i’ being a property value consistent with both statements.

If a statement ensemble contains a target clabject *v* and another target clabject *w* that is a direct or indirect super-clabject of *v*, then the statement with target clabject *v* is meaningless (can be deleted without effect). To avoid such situations, statements obey an additional integrity constraint: Target clabjects in a statement ensemble are orthogonal, that is, they are not in a direct or indirect generalization relationship (line 5:21). A violation of this integrity check should not be regarded as a model inconsistency but rather issued to the modeler as a warning.

#### Examples

The usage of DDM-FL for expressing clabject generalization with abstract super-clabjects and abstract statements is exemplified in Listing 6, which represents property engine (line 6:1) together with relevant parts of clabject hierarchies ProductHierarchy (line 6:4) and ProductComponentHierarchy (line 6:32) from the example shown in Fig. 10. Vehicle (line 6:7) is an abstract instance of Product. Car (line 6:9) is a concrete specialization of Vehicle; the indirect instantiation relationship to Product is not explicitly represented but derived. AcmeS28i at line 6:16 comes with an example of multiple generalization, it specializes LuxuryCar and SportsCar. A SportsCar has a PowerfulEngine as engine (line 6:13). An AcmeS22i car, a specialization of SportsCar, has a FuelEfficientEngine as engine  6:15.

A query on the range or value of property engine with AcmeS22i (or one of its instances) as source will only retrieve A2200i (or one of its instances) as consistent target, since it is the only engine that is both a specialization of PowerfulEngine and FuelEfficientEngine (see line 6:41).

**Fig. 11:**
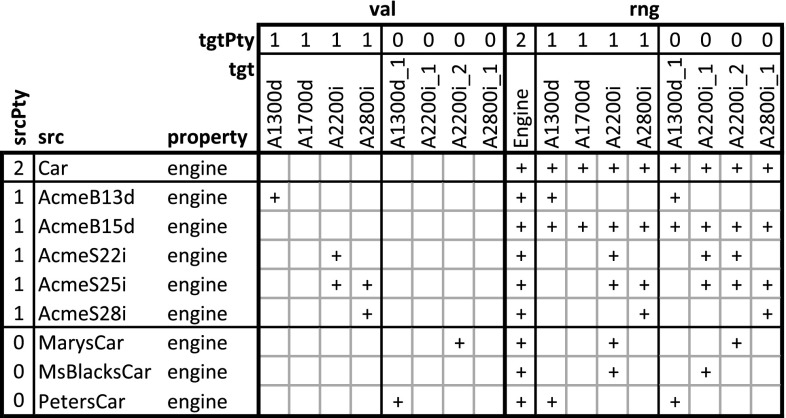
Compact representation of the results of querying the multi-level model in Listing 6 for the value and the range of property engine, restricted to concrete clabjects. Since range and value are bidirectional, this matrix also gives the range and value for property engine.inv

Figure 11 shows the results of queries on the range (see line 6:53) and the value (line 6:52) of property engine, restricted to concrete clabjects.

## Specialization hierarchies of deep properties

In this section, we extend DDM with deep property specialization. For this purpose, we adapt the principles of subsetting of association ends and specialization of associations (as known from the UML) and of property hierarchies (as known from OWL) for multi-level modeling.

### Background: property specialization in two-level models

Properties mainly come in two forms, namely as attributes (aka: data properties) and as association ends (aka: object properties). As discussed before, in this paper we do not distinguish between attributes and association ends.

Property specialization, as understood here, is similar to subsetting of association ends and specialization of associations in the UML. We do not distinguish between subsetting and specialization because they are semantically equivalent, as pointed out by Costal et al. [13].

A structural difference between subsetting and specialization is that the former is specified for an association end and the latter is specified for an association. This difference is of no interest in our setting, because an association is represented by a property and its implicitly introduced inverse property, and a specialization of a property implicitly introduces a specialization of the inverse property.

Subsetting of association ends and specialization of associations are arguably not used very frequently by UML modelers. This is in stark contrast to knowledge representation and ontology engineering, with specialization hierarchies of properties being one of the core modeling constructs of OWL.

Property specialization is a relationship between two properties, one in the role of the sub-property and the other in the role of the super-property, and has the following meaning: The domain of the sub-property must be the same or a subset of the domain of the super-property; the range of the sub-property must be the same or a subset of the range of the super-property. A value of the sub-property is always also a value of the super-property. The values of the sub-property must thus also be in the active range of the super-property. A range refinement on the super-property thus also affects the active range of the sub-property.

### Specialization of dual deep properties

The meaning of property specialization in two-level modeling should in principle also apply to specialization of dual deep properties. At the schema level, the domain and range refinements introduced with a deep property also apply to its specializations. At the instance level, values of the sub-property are also values of the super-property.

A specialization relationship connects a deep property in the role of *sub-property* with another deep property in the role of super-property. The sub-property’s source and target must be in the super-property’s domain and range, respectively. The sub-property does not specify its source potency and target potency but derives it from the super-property. A property may have multiple super-properties.

**Fig. 12:**
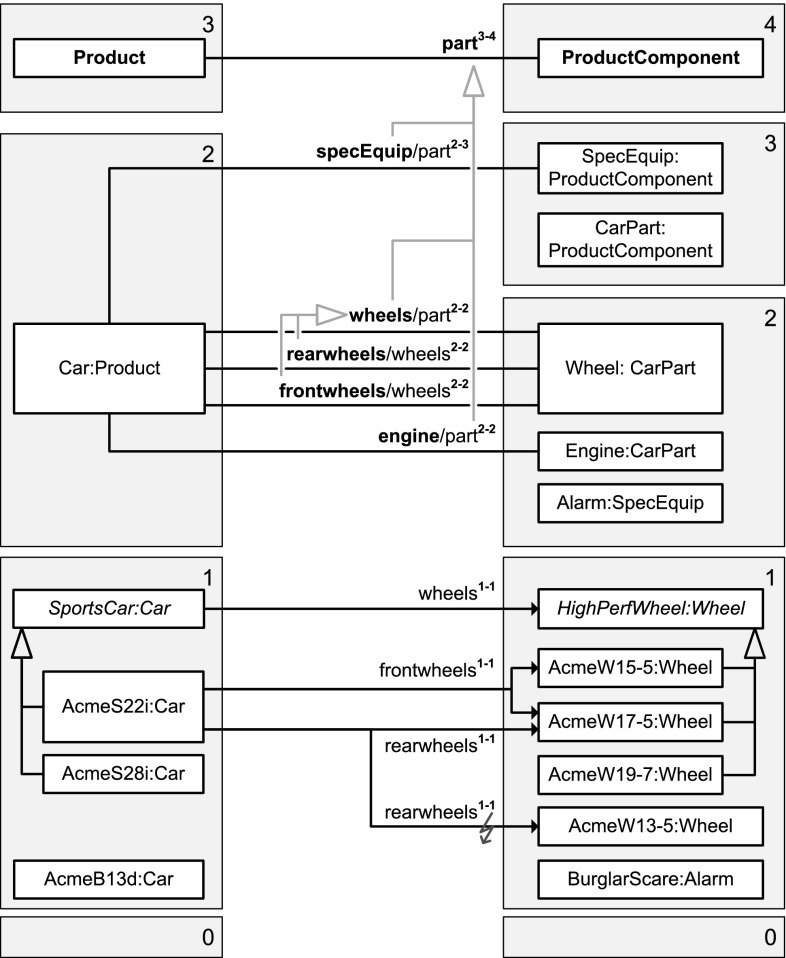
Specialization of multi-level properties. The declaration of sub-properties is shown by the name of the sub-property followed by a slash followed by the name of the super-property. The specialization is additionally highlighted by shaded arcs from sub-property to super-property. Introduced conflicts (depicted by a bolt symbol) are detected and resolved

Specialization hierarchies of deep properties are orthogonal to the range refinement and instantiation down the multi-level domain and multi-level range of a property. They provide an additional abstraction dimension and span all the different levels of a property.

#### *Example 32*

(Deep property specialization—Fig. 12) Property part is introduced between Product and ProductComponent with source potency 3 and target potency 4. The previously introduced properties specEquip and engine are now declared as specializations of part. A further sub-property of part is the wheels property which is introduced at product category Car with category Wheel as target. Source potency 2 and target potency 2 of property wheels are derived from the source potency (3) and the target potency (4) of property part and the number of instantiation steps between Car and Product (1) and between Wheel and ProductComponent (2). Property wheels is further specialized by properties frontwheels and rearwheels. Sub-properties frontwheels and rearwheels are, like property wheels, introduced between clabjects Car and Wheel and allow to make separate statements concerning rear wheels and front wheels at the different levels. The specialization hierarchies of properties provide an additional abstraction dimension in that one can separately read and write, e.g., for clabject AcmeS22i, value and range of the properties frontwheels and rearwheels or their super-property wheels or, in turn, the super-property part.

One aspect of property specialization hierarchies is that they allow to refine the range for a sub-property without affecting the range of the super-property. This is of particular importance when dealing with incomplete knowledge, especially when dealing with knowledge that is available at different levels of detail.

#### *Example 33*

(Parallel refinement and instantiation of super- and sub-properties—Fig. 12) The ACME product knowledge base contains rather strict range restrictions for the allowed wheel types of particular car models, while other aspects, such as the allowed types of special equipment are rather under-constrained. DDM with property hierarchies supports such multi-granular specification of models. Abstract class SportsCar refines the range of property wheels to HighPerfWheel. This range refinement affects front wheels and rear wheels of sports cars. From the different high-performance wheel models, sports car model AcmeS22i may use only wheel models AcmeW15-5 and AcmeW17-5 as front wheels and is restricted to wheel model Acme17-5 as rear wheels. The statement with AcmeW13-5 as target is in conflict with the above statement that SportsCars only have HighPerformanceWheels and is disregarded when querying range or values of property rearwheels.

### Extending DDM-FL with property specialization

Listing 7 extends the F-Logic formalization/implementation with property specialization. A property (class P) may additionally have multiple super-properties, and this is expressed by method super with unrestricted cardinality (see line 7:1).
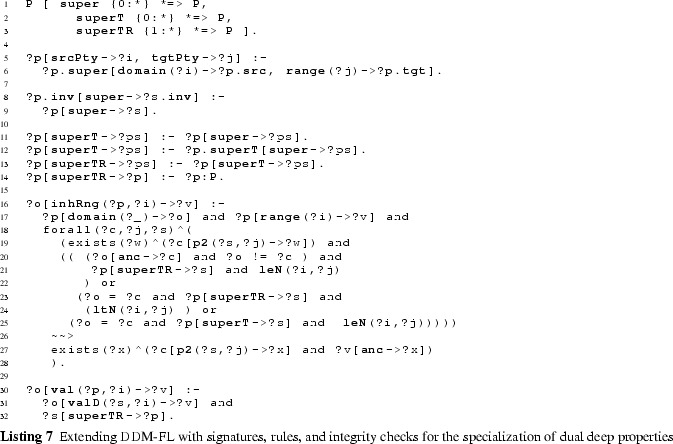


In derivation rules, one sometimes needs the transitive and the transitive reflexive closure of the super-properties of a property which are provided by derived methods superT and superTR (which are declared at lines 7:2-7:3 and defined at lines 7:11-7:14).

When a property *p* has a super-property *s*, then the inverse of *p* has the inverse of *s* as a super-property (see rule at line 7:8).

The potencies of a sub-property need not be asserted, and they are inherited from the super-property. The source/target potency of a sub-property is the number of the level of the domain/range of the super-property at which the source/target clabject of the sub-property resides (see rule at line 7:5).
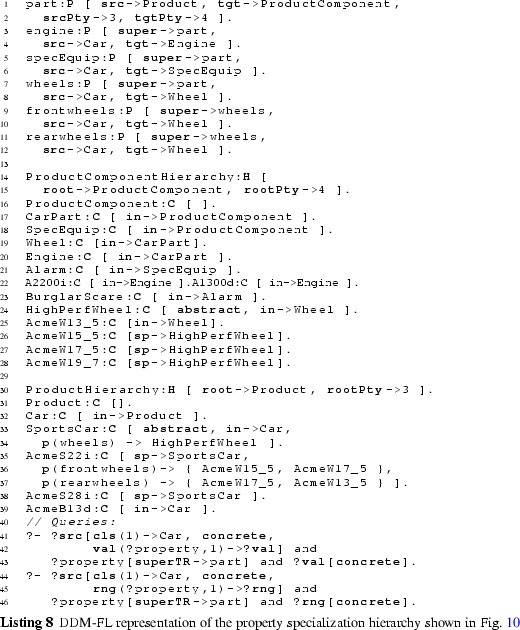

**Fig. 13:**
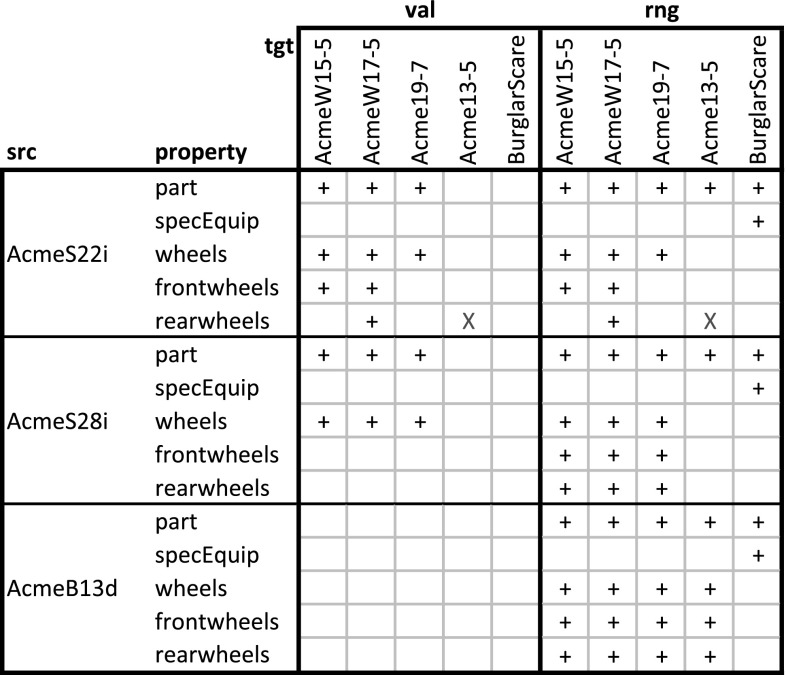
Compact representation of the results of querying the multi-level model in Listing 8 for the value and the range of property part and its sub-properties for concrete car models. ‘$+$’ indicates the existence of a property instance (either rng or val), ‘$\times$’ indicates an inconsistent target that has been discarded from the result

At the schema level, a range refinement imposed on a property also applies to its sub-properties. This is reflected in DDM-FL by a redefinition of method inhRng (superseding the definition in Listing 3) by the rule at lines 7:16-7:28. The general idea that the inherited range is the intersection of all range refinements introduced by superordinate statements remains unchanged with the addition that statements on super-properties are also considered as superordinate statements. The redefinition of inhRng also affects other methods introduced in Listing 3, namely rngD, actRng, valD, and val, which now all produce results that are consistent with range refinements introduced with super-properties.

At the instance level, a value of a property is also a value of its super-properties. This is expressed by the rule at line 7:30 which extends the definition of the previously introduced method val (see Listing 3) to also include consistent values of the sub-property as consistent values of the super-property.

#### Examples

The representation of property specialization in DDM-FL is exemplified in Listing 8 together with queries on the value (line 8:41) and the range (line 8:44) of sub-properties of part, only considering concrete clabjects at level 1 of the multi-level range. Figure 13 gives a compact representation of the results of these queries.

## Dual deep cardinality constraints

In this section, we extend DDM with deep cardinality constraints both regarding the schema facet (range) and the instance facet (values) of properties. Value cardinality constraints restrict the number of property values; they are evaluated over the instance facet of a property. Range cardinality constraints are quite different, and they restrict the number of clabjects in the range of a property; thus, they are evaluated over the schema facet of a property.

### Modeling with dual deep cardinality constraints

Cardinality constraints in DDM only consider concrete clabjects. Abstract clabjects are regarded as mere abstractions of concrete clabjects without an own identity,

A *deep cardinality constraint*  is specified for the instance facet (value) or the schema facet (range) of a deep property, a source clabject (within the domain of the property), a target clabject (within the range of the property), a source potency specifying the to-be restricted level of the multi-level domain and a target potency specifying the to-be restricted level of the multi-level range. Cardinality constraints can be specified for both ends of an association, for a property as well as for its inverse.

**Fig. 14:**
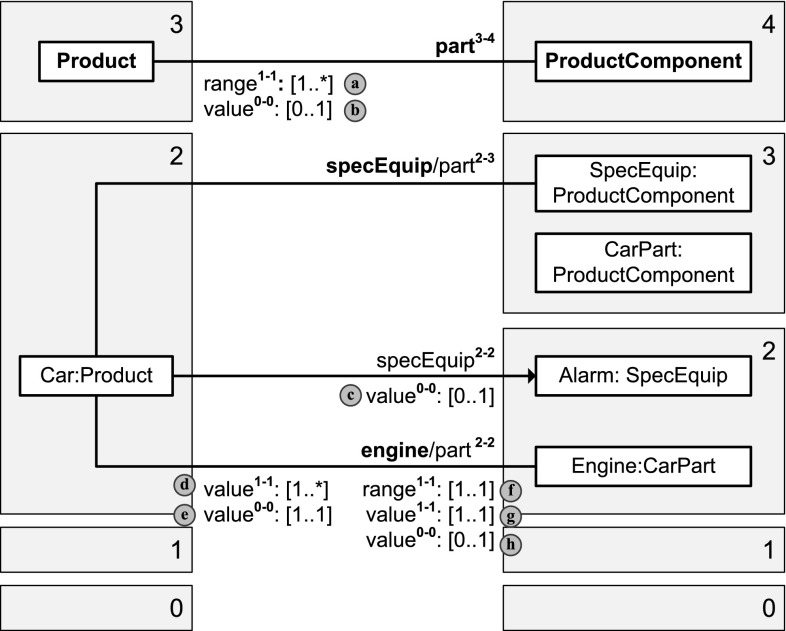
Example multi-level cardinality constraints over property range and property values. The different constraints, labeled with *letters* (a)–(h), are explained below

In the UML-like notation (see example in Fig. 14), cardinality constraints are shown at either end of the association: constraints shown at the same end as the property name (e.g., constraints labeled (c), (f), (g), (h)) restrict the cardinality of the property, and constraints shown at the opposite end (e.g., constraints labeled (a), (b), (d) and (e)) restrict the cardinality of the inverse property.

A *deep functional value constraint* specifies that clabjects at the given domain level have at most one property value at the given range level. A *deep mandatory value constraint* likewise specifies the existence of at least one property value at the given range level.

#### *Example 34*

(Deep functional and mandatory value constraints—Fig. 14) The deep model fragment shown in Fig. 14 contains three deep functional value constraints. The cardinality constraint labeled (c) expresses that between cars and alarm systems at the individual-to-individual level (expressed by dual potency $^{(0,0)}$), for one car, there may only be one concrete special equipment value. This means that every individual car represented in the multi-level model may only have one individual alarm as special equipment. Constraint (g) is a mandatory and functional value constraint at the model-to-model level$^{(1,1)}$ of property *engine*, which restricts car models to have exactly one engine model as value of property engine. Constraint (h) is a functional value constraint at the individual-to-individual level$^{(0,0)}$ of property engine and expresses that every individual car has at most one individual engine as engine.

Deep cardinality constraints may also be expressed on inverse properties and be evaluated—with the same semantics as cardinality constraints on non-inverse properties—over value and range of the inverse property.

#### *Example 35*

(Deep functional and mandatory value constraints on inverse properties—Fig. 14) The mandatory constraint (d) between Engine and Car at the model-to-model level$^{(1,1)}$ of the inverse property of engine expresses that every car engine model is engine of at least one car model. The mandatory and functional constraint (e) between Engine and Car at the individual-to-individual level$^{(0,0)}$ expresses that every individual car engine is engine of exactly one car individual. In the context of the ACME product knowledge base, engines are of interest only as part of cars and should be discarded if not part of a car.

A *qualified cardinality constraint* is a deep cardinality constraint, where the target clabject is not the root of the property’s range. This allows to express multiplicities that only affect a sub-hierarchy of the properties range.

#### *Example 36*

(Qualified cardinality constraints—Fig. 14) The functional constraint (c) on property specEquip between Car and Alarm is a qualified cardinality constraint. It restricts individual cars to have at most one individual alarm system as special equipment (but an individual car may have a lot of other special equipment individuals, as long as they are no alarm systems).

A *refined cardinality constraint* is a cardinality constraint, where the source clabject is not the root of the property’s domain. It is checked only for clabjects that are descendants of the source clabject of the constraint. Refined cardinality constraints can be qualified or unqualified.

A *cardinality constraint over a sub-property* only restricts the cardinality of the sub-property but not the cardinality of its super-property. In the opposite direction, since every value of a sub-property is also a value of its super-properties, a *cardinality constraint over a super-property* also indirectly affects its sub-properties.

#### *Example 37*

(Deep cardinality constraints and property specialization—Fig. 14) Properties specEquip and engine are sub-properties of part. The functional and mandatory value constraint (g) at the model-to-model level$^{(1,1)}$ only affects property engine but not its super-property part, a car model must not have more than one engine model as engine, but it can have more than one engine model as value of part (note, the given example is under-constrained, an additional functional value constraint at the model-to-model level$^{(1,1)}$ between Product and Engine would disallow such situations). The functional value constraint (b) at the individual-to-individual level$^{(0,0)}$ on the inverse of part affects specEquip and engine insofar that an individual product component already used as part of one product individual may not be used as engine or special equipment of another product individual.

A *mandatory range constraint*  specifies for clabjects at a specific level of the domain that they have at least one possible target at the specified level of the range (i.e., at least one clabject at that level must be in the range). The enforcement of mandatory range constraints should avoid situations where the refinement of the range at various source clabjects (for example at multiple superclasses of the clabject at hand) leads to an empty range. A *functional range constraint* specifies for clabjects at a specific level of the domain that they have at most one target clabject at the specified level of the range. A functional range constraint can be regarded as representing an obligation of the modeler to model range refinements at a specific granularity.

#### *Example 38*

(Mandatory and functional range constraints—Fig. 14) The constraint labeled (f) represents a mandatory range constraint and a functional range constraint for level 1 of the domain and level 1 of the range of property engine. The mandatory constraint specifies that each car model must have at least one car engine model as range of property engine. The functional range constraint specifies that each car model must have at most one car engine model in its range. That is, the modeler is obliged to refine, for each car model, the range of property engine to a single engine model.

Using inverse properties, range constraints can likewise be defined in the opposite direction.

#### *Example 39*

(Range cardinality constraints on inverse properties—Fig. 14) The constraint labeled (a) between Product and ProductComponent on the inverse of property part at the *model-to-model* level$^{(1,1)}$ specifies that each product component model (i.e., each clabject with potency 1 in the product component hierarchy) is in the range of property part of at least one product model. The rationale behind this constraint is that the primary purpose of the representation of product components in the ACME enterprise database is the detailed description of products, and if a clabject is representing a product component model that cannot be used as part of a product in the system, then this clabject is not needed and should be discarded from the knowledge base.

### Specifying and checking cardinality constraints in DDM-FL


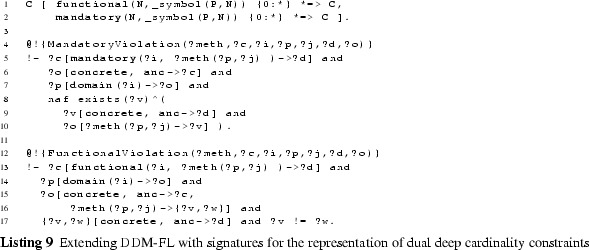


Deep *functional* and *mandatory* constraints over the schema facet (rng) or the instance facet (val) of a deep property or its inverse are specified using methods functional and mandatory of class C (see signatures at lines 9:1-9:2). A cardinality constraint is specified between a source clabject *c* and a target clabject *d* with arguments specifying the property *p* together with the level *i* of the domain and the level *j* of the range of the property and the property facet (val or rng) that should be restricted by the constraint. It constrains the number of links with source potency *i* and target potency *j* between descendants of *c* and descendants of *d*.

#### *Example 40*

**Fig. 15:**
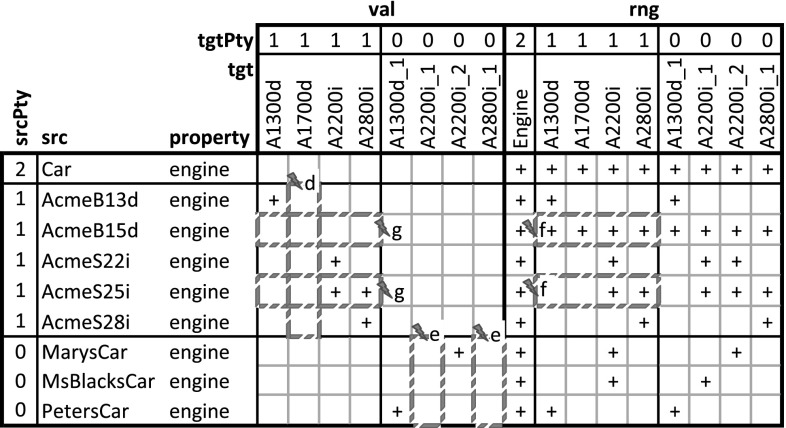
Evaluation of cardinality constraints of Fig. 14 (also represented in Listing 10) over value and range of property engine based on the statements modeled in Fig. 10 (also represented in Listing 6). Cardinality constraint violations are indicated by a thunderbolt symbol together with a letter identifying the cardinality constraint in Fig. 14

(Deep cardinality constraints in F-Logic) Listing 10 shows how the example cardinality constraints introduced in the previous subsection are expressed in F-Logic. For example, line 10:7 represents the mandatory and functional range constraint on the model-to-model level$^{(1,1)}$ of property engine between Car and Engine (labeled (f) in Fig. 14). Figure 15 exemplifies the evaluation of these cardinality constraints.


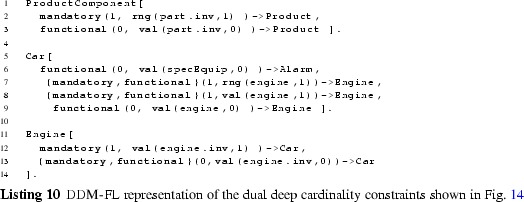

Note that the property facet (val or rng) together with property *p* and target potency *j* is combined into a function-valued argument, e.g., val(engine,0). The generic definition of functional and mandatory in DDM-FL would allow to specify cardinality constraints not only on val or rng but also on other methods with a similar signature, such as actRng.

A violation of a *mandatory constraint* (lines 9:4-9:10) for value or range (?meth) of property *p* between source clabject *c* and target clabject *d* for source potency *i* and target potency *j* (line 9:5) occurs, if a concrete ancestor *o* of *c* (line 9:6) at level *i* of *p*’s domain (line 9:7) has no concrete descendant *v* of *d* (line 9:9) as value or range, respectively, with target potency *j* for property *p* (line 9:10).

A violation of a *functional constraint* (lines 9:12-9:17) for value or range (?meth) of property *p* between source clabject *c* and target clabject *d* for source potency *i* and target potency *j* (line 9:13) occurs, if a concrete descendant *o* of *c* (line 9:15) at level *i* of the domain of *p* (line 9:14) has two targets *v* and *w* for property *p* at target potency *j* (line 9:16) which are both concrete descendants of *v* (line 9:17).

## Related work

Multi-level modeling offers more elegant solutions to many practical modeling situations than traditional, two-level modeling. Multi-level modeling approaches liberate modelers from the confinements of traditional metamodeling with model and metamodel, class and metaclass. The *clabject* [2], with its class and object facets, becomes the central notion. The number of metalevels is arbitrary and unconstrained, with each clabject possibly serving as the class for clabjects at a lower (meta-)level while, at the same time, instantiating a clabject from a higher metalevel. Common modeling patterns that typically benefit from a multi-level approach include the type-object pattern, the dynamic addition of features, and the configuration of relationships (see [33]). The type-object pattern refers to the dynamic creation of types at run time. Besides the type-object pattern, the need for dynamic addition of features to types frequently arises. Furthermore, certain use cases require the dynamic definition of relationships. In the DDM approach, self-describing objects provide the support for dynamic addition of features and configuration of relationships.

*Telos* [34] and its implementation *ConceptBase* [22, 23] allow for arbitrary-depth metamodeling. An attribute proposition may be instantiated recursively many times, thereby acting as referential integrity constraint for all its direct and indirect instances. The Telos axiomatization, however, does not specify a mechanism for the definition of the depth of property characterization. Furthermore, as opposed to DDM, Telos does not support the modeling of self-describing objects nor the flexible instantiation of properties. The instantiation of an attribute proposition always comes in conjunction with an instantiation of source and target. Likewise, Telos lacks a mechanism for the definition of deep cardinality constraints as well as a mechanism akin to property specialization, which is orthogonal to property instantiation by statements at different levels. On the other hand, Telos, just like DDM, does not distinguish between associations and attributes.

Unlike DDM, Telos does not distinguish properties from statements. Rather, in Telos, every property is also a statement. Furthermore, Telos treats explicit instantiations as regular statements, i.e., as attributes of objects; DDM differs in this respect. The lack of potencies in Telos do not prevent the definition of statements that cross-abstraction levels, e.g., from a metaclass to an individual. Such a statement, however, may only be instance of another statement if that other statement’s source and target are both one metalevel above the instantiating statement’s source and target. Finally, DDM’s generalization of properties is comparable to Telos property generalization. But, since Telos is ignorant of the concept of potency, generalization has no impact on potency in Telos, as opposed to the generalization of properties in DDM.

*VODAK* [26] was among the first systems that introduced what was later called deep instantiation/characterization [17, 29]. The VODAK system realizes deep instantiation through the organization of objects into types and, in parallel, into classes and metaclasses. Metaclasses define own types, instance types and instance-instances types, similar in essence to the *power type* design pattern [17]. In VODAK, type instantiation occurs between an object and its class’ instance type; type instantiation is inferred from the functional association of an object to a class in order to provide for a lossless and redundancy-free representation of functional data dependencies according to database design theory. As opposed to DDM, VODAK captures the semantics of relationships between objects at different metalevels in a behavioral manner, using methods attached to types rather than implicit constraints.

Potency-based metamodeling allows for a class at one (meta-)level to define features of instances several levels below. To each feature, the class assigns a potency which indicates the metalevel that the feature characterizes, with instances at the respective level providing the actual values for the feature [5]. The traditional potency-based metamodeling approach [6] assumes strictness. In a strict metamodeling framework, instantiation is the only relationship to cross-level boundaries. Any other relationship occurs between objects at the same metalevel [7]. Following a data modeling agenda, the DDM approach relaxes the strictness condition by distinguishing between source and target potencies.

Model-driven engineering (MDE), which typically relies on two metalevels, namely model and metamodel, may benefit from the introduction of an arbitrary number of metalevels. Several MDE frameworks support multi-level modeling. *DeepJava* [28] extends the Java programming language with multiple metalevels and potencies, effectively abandoning two-level programming with class and object. *Melanee* [3, 4, 24] provides support for the design of domain-specific languages. *Nivel* [1] defines a potency-based metamodeling language with a formal specification of the language semantics. *MetaDepth* [30] presents a multi-level modeling framework with support for the definition of constraints and derived attributes.

The MetaDepth multi-level metamodeling framework employs richer semantics for potencies than previous approaches toward potency-based metamodeling, thereby extending multi-level modeling for more practical applications [31, 32]. In MetaDepth, several models exist which represent different viewpoints on a system. Each model belongs to some metalevel as indicated by the model’s potency. A clabject may refer to another clabject within the same model. *Deep references* relate clabjects at different metalevels, thereby realizing a cross-level relationship other than instantiation, loosening the strictness of metamodeling. References and deep references in the MetaDepth framework are akin to properties in DDM.

The DDM approach incorporates many traits of MetaDepth geared toward increased modeling flexibility. In particular, DDM allows for property relationships between clabjects at different metalevels. MetaDepth realizes cross-level references via the definition of different models, whereas DDM introduces a target potency for deep properties. DDM also adopts a kind of leap semantics for property instantiation. In DDM, properties are not mediated, that is, not mandatorily instantiated at every metalevel. Rather, DDM allows for the refinement of range and the instantiation of values at every metalevel without forcing modelers to do so.

Rossini et al. [42] provide a formalization of deep metamodeling based on graph transformation using the Diagram Predicate Framework (DPF). The formalization receives an implementation in MetaDepth. The formalization distinguishes between single-potency and multi-potency semantics. Deep attributes with multi-potency semantics are instantiated at multiple metalevels. As in MetaDepth, deep instantiation with multi-potency semantics is usually mediated, as opposed to DDM which is more flexible in this regard.

Unlike MetaDepth and other related multi-level modeling approaches, which place the focus on software engineering, DDM aims primarily at data engineering. The main concerns of DDM are thus the modeling of domain and range of object properties, the querying of data objects, and the representation of asserted values as opposed to derived values. In particular, range, active range and (derived) values are important DDM concepts for data modeling not present in MetaDepth and other multi-level modeling approaches that focus on software engineering.

The data modeling focus of DDM suggests employing the abstract superclass rule which presents considerable advantages for object-oriented data modeling (see [19]). In particular, the abstract superclass rule alleviates the issues associated with multiple inheritance. Furthermore, the abstract superclass rule facilitates querying since each class also represents a set of objects.

*Materialization* [14, 40] realizes a multi-level conceptual modeling approach with a relationship that blurs the boundaries between aggregation and instantiation. Data objects may have a class facet and an object facet. Different types of attribute propagation in the course of materialization emulate the deep characterization pattern.

The *powertype*-based approach toward metamodeling also relies on the notion of clabject [17]. The instances of a powertype are sub-types of another object type, thereby providing metamodeling capabilities [38, p. 28]. The powertype, which is also part of the UML standard [39, p. 54], traces its origins to powersets in set theory [8]. The powertype pattern consists of “a pair of classes in which one of them (the powertype) partitions the other (the partitioned type) by having the instances of the former be sub-types of the latter” [17, p. 83]. A more recent approach [16] adopts a viewpoint on modeling and metamodeling based on the use of language rather than originating in set theory.

The multi-level object (m-object) combines the characteristics of deep instantiation, materialization, and powertypes [35]. An m-object defines a class for various, hierarchically ordered levels of abstraction while instantiating the top-level class. In DDM, each clabject has a class facet for each metalevel and an object facet for the top metalevel. In this respect, a clabject in DDM is similar to an m-object. In contrast to DDM and other potency-based deep modeling approaches, the m-object approach uses level names instead of potency numbers to refer to instantiation levels and supports the injection of intermediate levels for some part of a hierarchy. The m-object modeling approach, however, has no equivalents to clabject generalization, property specialization, and deep cardinality constraints, and also lacks the powerful query facilities of DDM.

Demuth et al. [15] identify consistency checking as an important aspect of multi-level modeling. The thus identified types of consistency checking in multi-level models include checking conformance of model elements with the linguistic metamodel, checking conformance of instances with ontological types, checking consistency of inheritance relationships between ontological types, and semantic constraint checking for objects at a specific metalevel. The DDM formalization in F-Logic contains rules for consistent specialization and type conformance as well as a mechanism for the definition of multiplicity, domain, and range constraints for relationships. This paper does not explicitly investigate the definition of additional semantic constraints for DDM models, e.g., that the list price of car models of a specific category must exceed a minimum amount of money. Modelers, however, may define additional semantic constraints over classes by using F-Logic and the DDM predicates. Demuth et al. [15] introduce a formalization of multi-level modeling based on the DesignSpace Core metamodel in order to support automated consistency checking. The DDM formalization in F-Logic allows modelers to employ the Flora-2 system in order to check consistency of a model. Demuth et al. [15] further stress the importance of consistency checking for multi-level model evolution. Again, for DDM models, modelers may employ the Flora-2 system in order to determine consistency of an intended change in the model.

The MLT multi-level theory [9] establishes ontological foundations for multi-level modeling. MLT applies the Unified Foundational Ontology (UFO), a reference ontology the main application area of which is conceptual modeling, to multi-level modeling. In particular, unlike UFO, MLT distinguishes not only between types and individuals but also considers types with types as instances. This extension of UFO for multi-level modeling improves semantic clarity of multi-level models.

The visual and precise metamodeling (VPM) framework [45] aims at a mathematical definition of visual metamodeling and follows a multi-level approach. The VPM framework has a model-driven architecture focus where the quality of the metamodels is of critical importance for model transformation. In VPM, the boundaries between model and metamodel are fluid. What is model may be metamodel in another context, type–instance relationships between models are handled dynamically, which presents advantages for model transformation. The VPM framework employs multi-level modeling in order to allow for the specialization of classes between models without being confined by the strict division of metalevels in MOF. Importantly, in VPM, model elements may be, at the same time, instance and specialization of a particular model element. VPM, however, has no equivalent to potency-based instantiation. DDM has a focus on data modeling and knowledge representation, the concept of potency-based deep instantiation being a central aspect.

The SKIF framework [18] introduces a definition of the semantics of the Knowledge Interchange Format (KIF). The SKIF framework employs the notion of *term* as the central concept, bearing similarities to the clabject-centric view of multi-level approaches. Although SKIF refers to *individuals* and *relations*, an individual is structurally indistinguishable from a relation, defined as a relation with an empty extension. The SKIF framework shows that first-order logic is sufficient to express such multi-level models.

F-Logic and the Flora-2 system provide the formal framework and modeling environment for DDM. The Flora-2 system has already proven its suitability for formalizing multi-level modeling [20]. A different approach [12] aims at providing a modeling kernel as a foundation for multi-level modeling, suitable for the representation of deep instantiation and powertype-based models. Unlike this previous formalization of multi-level modeling in F-Logic, DDM considers cross-level property relationships a core concern of the approach. Thus, in this paper, we present an extensive investigation of such properties.

## Conclusions

In this paper, we introduced DDM as a general purpose multi-level modeling approach with a focus on the requirements of conceptual data modeling. The DDM language constructs generalize core object-oriented modeling constructs—class, object, property, property value, association, link, association end refinement, multiple class/object generalization, property/association specialization, cardinality constraints—for multi-level modeling. In contrast to strict multi-level modeling approaches, different clabject hierarchies in the same model may come with varying numbers of instantiation levels. Properties and their statements may flexibly connect clabjects at different levels.

We introduced DDM along examples from a multi-level enterprise database. Similar use cases and settings can be found in manufacturing enterprises that organize multiple product models in so-called product life-cycle management (PLM) systems. These systems describe in detail the properties of products, their parts, the machinery for their production, and the production steps. Such systems need to be integrated with the companies’ operational systems, e.g., ERP systems that manage the actual resources, the orders, the customer base, and so forth. Whereas ERP systems and operational systems focus on instance data, PLM systems focus on models, which act as classes in the operational systems. Both must be integrated to create a consistent view of the enterprise, i.e., the integrated conceptual data model of the ANSI/SPARC architecture. A flexible multi-level modeling approach like DDM allows to make associations between the two types of systems independently of their modeling levels, and this is necessary since neither is just the schema for the other. Experience from industry projects [44] suggests particular adequacy of multi-level modeling for IT ecosystem integration.

The formalization of DDM in F-Logic/Flora-2 (DDM-FL) constitutes a proof-of-concept implementation of the approach. The DDM-FL system provides consistency checking and flexible query facilities to the modeler, especially for querying property values (instance facet) and range of properties (schema facet), derived from the multi-faceted statements at the various levels of the multi-level model/database.

The list of ongoing and future work based on the DDM approach includes:

- Multi-level modeling of n-ary associations and association classes.
- Modeling of derived properties, e.g., avgAge, of clabjects.
- Modeling and specification of operations (methods) of clabjects along with life-cycle models in the spirit of multi-level business processes [43].
- Adapting and interfacing DDM to standard modeling and data representation techniques and tools.

## Appendix: Complete formalization of DDM in F-Logic

The formalization of DDM in F-Logic, which was developed and tested with version 0.99.5 of Flora-2, together with examples, is available at http://multilevelmodeling.net/. The Flora-2 system is available at http://flora.sourceforge.net/. The following code listing contains the complete formalization/implementation of DDM-FL, merging the code fragments introduced in this paper.

## Footnotes

1
http://flora.sourceforge.net/.

2
http://xsb.sourceforge.net/.

3
http://flora.sourceforge.net/docs/floraManual.pdf.
